# High richness of ectomycorrhizal fungi and low host specificity in a coastal sand dune ecosystem revealed by network analysis

**DOI:** 10.1002/ece3.1881

**Published:** 2015-12-29

**Authors:** Alice Roy‐Bolduc, Etienne Laliberté, Mohamed Hijri

**Affiliations:** ^1^ Département de Sciences BiologiquesInstitut de Recherche en Biologie VégétaleUniversité de Montréal4101 Sherbrooke EstMontréalQuébecCanadaH1X 2B2

**Keywords:** 454 sequencing, coastal sand dunes, ectomycorrhizal fungal community, host preference, network analysis, plant–fungal interactions

## Abstract

Ectomycorrhizal (EM) fungi are ubiquitous in temperate and boreal forests, comprising over 20,000 species forming root symbiotic associations with Pinaceae and woody angiosperms. As much as 100 different EM fungal species can coexist and interact with the same tree species, forming complex multispecies networks in soils. The degree of host specificity and structural properties of these interaction networks (e.g., nestedness and modularity) may influence plant and fungal community assembly and species coexistence, yet their structure has been little studied in northern coniferous forests, where trees depend on EM fungi for nutrient acquisition. We used high‐throughput sequencing to characterize the composition and diversity of bulk soil and root‐associated fungal communities in four co‐occurring Pinaceae in a relic foredune plain located at Îles de la Madeleine, Québec, Canada. We found high EM fungal richness across the four hosts, with a total of 200 EM operational taxonomic units (OTUs), mainly belonging to the Agaricomycetes. Network analysis revealed an antinested pattern in both bulk soil and roots EM fungal communities. However, there was no detectable modularity (i.e., subgroups of interacting species) in the interaction networks, indicating a low level of specificity in these EM associations. In addition, there were no differences in EM fungal OTU richness or community structure among the four tree species. Limited shared resources and competitive exclusion typically restrict the number of taxa coexisting within the same niche. As such, our finding of high EM fungal richness and low host specificity highlights the need for further studies to determine the mechanisms enabling such a large number of EM fungal species to coexist locally on the same hosts.

## Introduction

Ectomycorrhizal (EM) fungi are plant symbionts colonizing the roots of many tree and shrub species, for example, conifers in the Pinaceae such as *Pinus*,* Picea*, and *Larix*. These fungal symbionts, ubiquitous in forested ecosystems of temperate and boreal biomes, are crucial for the growth and survival of their hosts as they enhance nutrient and water uptake and protect plant roots from infection by pathogens (Smith and Read 2008). Ectomycorrhizal communities generally contain high numbers of fungal taxa associating with a low diversity of host plants (Tedersoo et al. 2014; van der Heijden et al. 2015). Northern forests, generally poor in tree species, can support several hundreds of EM taxa (Trappe 1977; Horton and Bruns 2001). Furthermore, as much as 100 different EM fungal species can interact locally with one tree species in a single monospecific stand (Allen et al. 1995), and over 15 EM fungi can be found in association with a single individual (Saari et al. 2005). The structural properties of these complex species interaction networks can be characterized using bipartite network analysis, where the different species are represented as nodes belonging to two classes (EM fungi or host plant) that can be linked depending on the strength or frequency of the interspecies interactions (e.g., Bahram et al. 2014). In such ecological networks, species may display various levels of specialization, which would result in different network structural properties.

The two most commonly characterized network structural properties are nestedness and modularity. Nestedness is a measure of the hierarchical organization of interactions (Bascompte et al. 2003). In nested networks, specialized species are mainly associated with the generalist species of the other class but not with other specialists (Bascompte et al. 2003). High nestedness is thought to enhance the diversity and resilience of ecological communities (Burgos et al. 2007; Chagnon et al. 2012), particularly by reducing interspecific competition and facilitating species coexistence (Bascompte et al. 2003; Bastolla et al. 2009). Modularity, on the other hand, is a measure of reciprocal specialization. It evaluates the presence of modules, that is, subgroups of strongly interacting species. The modular organization of complex networks, resulting from functional complementarity and coevolutionary dynamics, is believed to increase overall network stability particularly by containing the effects of perturbations within compartments and therefore buffering communities against secondary extinctions following disturbance (Guimera and Amaral 2005; Stouffer and Bascompte 2011).

The architecture of plant–fungal species interaction networks remains poorly studied, mainly because of the technical limitations to the accurate taxonomic description of microbial communities. The recent developments in next‐generation sequencing techniques have greatly increased our ability to characterize soil microbial community composition and diversity (Nilsson et al. 2011; Tedersoo et al. 2014). Still, we know little about underground plant–fungal network architecture in most ecosystems. The structure of mutualistic networks (e.g., plant–pollinator networks) has been commonly described as highly nested (e.g., Bascompte et al. 2003; Burgos et al. 2007; Thebault and Fontaine 2010). The general expectation for plant–fungal mutualistic associations would therefore be a nested network organization. However, recent studies on the structure of belowground plant–fungal networks observed various results depending on environmental context and mycorrhizal type. For example, Jacquemyn et al. (2015) observed a high degree of specialization and modularity between orchids and their associated orchid mycorrhizal fungi. By contrast, network analysis of arbuscular mycorrhizal (AM) fungal communities showed significant nestedness (Chagnon et al. 2012; Montesinos‐Navarro et al. 2012). With regard to EM fungal communities, Bahram et al. (2014) analyzed ten EM plant–fungal interaction networks and found significant negative relative nestedness (i.e., “antinestedness”) and, in some cases, significant levels of modularity.

Differences in the ecology of the distinct types of mycorrhizal symbiosis may explain the differences observed in network properties. Arbuscular mycorrhizas are formed by the association of Glomeromycota fungi, a phylum currently comprising only 244 described species (Schüßler and Walker 2010), with a high diversity of hosts – around 200,000 plant species (Brundrett 2009). Ectomycorrhizal and orchid mycorrhizal symbioses are more balanced with regard to number of fungi and host plants, with, respectively, 20,000 and 25,000 fungal species associating with around 6000 and 20,000–35,000 plant species (reviewed in van der Heijden et al. 2015). Hence, we could expect a higher potential of preferred associations in EM fungi compared to AM fungi.

A few studies have characterized soil fungal communities in coastal sand dune ecosystems, but those have mainly focused on AM fungi (Koske and Halvorson 1981; Corkidi and Rincón 1997; Koske and Gemma 1997; Kowalchuk et al. 2002; Błaszkowski and Czerniawska 2011; etc.). To our knowledge, EM communities and multispecies network have not previously been described in coastal dune ecosystems. In this study, we used high‐throughput sequencing to characterize soil and root‐associated EM fungal communities in four co‐occurring Pinaceae tree species in a relic foredune plain. We also used network analysis to further describe network structure and specificity of associations, and to compare network properties with that of previous studies in different systems.

Previous studies in Northern Hemisphere ecosystems observed overlapping communities, suggesting that most EM fungi have multihost habits (Horton and Bruns 1998; Cullings et al. 2000; Horton and Bruns 2001; Kennedy et al. 2003). However, recent work relying on high‐throughput sequencing was able to detect different degrees of association preferences (e.g., Ishida et al. 2007; Morris et al. 2008; Aponte et al. 2010; Tedersoo et al. 2010; Murata et al. 2013), and tree species identity is increasingly recognized as a key factor shaping EM fungal communities (Smith et al. 2009). As such, we expected to observe – in addition to some multihost fungi – several specialists preferentially interacting with a single plant, resulting in a moderate to high effect of host identity on the structure of EM communities. We hypothesized that this host effect would translate into the absence of nested patterns and significant levels of modularity, as highlighted by Bahram et al. (2014) in other forested ecosystems dominated by EM trees.

## Material and Methods

### Study area and site description

Our study system is a relic foredune plain known as “Les Sillons” located within the Îles de la Madeleine, an archipelago situated in the southern Gulf of St. Lawrence in Québec, Canada (47°23′N, 61°52′W). Îles de la Madeleine is characterized by a maritime cold temperate climate. Mean annual temperature on the islands is 4.5°C and annual precipitation sums to 987 mm, of which approximately 30% falls as snow (Houle 2008). “Les Sillons” covers a crescent‐shaped area of 10.6 km^2^ and is composed of a series of shore‐parallel ridges. Resulting from seaward growth, the system is a sandy depositional barrier that accumulated during the Holocene and now connects two bedrock islands (Giles and King 2001). Soils are mainly sandy.

“Les Sillons” includes a succession of habitats from the coast, ranging from the beach to mobile dunes and heathlands, and then to forests (on dune crests) and wetlands (in dune swales). The mobile dunes are characterized a plant community that is largely dominated by *Ammophila breviligulata*, the American beachgrass, as well as a few herbaceous plant such as *Artemisia stellaria* and *Fetusca rubra* in areas that are not directly exposed to wind and salt spray. Heathlands are dry habitats which are dominated by shrubs such as *Myrica pensylvanica*,* Juniperus communis,* and *Spirea alba,* and these transition into black spruce, fir, and pine forests in the older dunes. The inter‐ridge swales harbor a diverse array of vegetation, including several Ericaceae, *Sphagnum*, and *Carex* species. Details about the study system and its soil fungal communities are described in more detail in Roy‐Bolduc et al. (2015).

Our sampling was concentrated in a 3‐ to 4‐km‐long portion of the forested dunes, which are roughly parallel to the coastline. The forest canopy is composed of mixed and distinct stands of Pinaceae (e.g*., Abies balsamea, Picea mariana, Picea glauca, Pinus banksiana,* and *Pinus mugo*), and the understory includes shrubs such as *Myrica gale* and *Chamaedaphne calyculata*. The forest floor is often covered with lichens (*Cladonia* spp.). Although the area was never intensively exploited for timber, the forest is mainly secondary because of small‐scale punctual logging, natural fires, and anthropogenic disturbances that occurred during the construction of the highway and the development of the electricity network over the last century. We sampled fine roots (<2 mm diameter) and associated soil of four of the codominant tree species of this dune system, including two non‐native species (*Pinus banksiana* and *Pinus mugo*) used in plantations for dune stabilization in the 1940–1960 period (O'Carroll 1998) and two native and naturally occurring species (*Picea mariana* and *Abies balsamea*). *Pinus banksiana* is commonly found in North American boreal forests, but was not originally present on the Îles de la Madeleine. *Pinus mugo*, a small tree native from high elevation habitats in Europe, is well adapted to dry soils and low nutrient concentrations. Eight replicates of root and soil samples were sampled in August 2010 for each species in monospecific stands of at least 10 × 10 m. These stands were randomly selected within the forested dune but were at least 100 m from each other to minimize spatial autocorrelation. Roots identity was confirmed by tracing roots back to the main trunk. Soil samples were composed of a mixture of six 0‐25 cm deep soil cores collected randomly within a 1 m^2^ plot located around the tree trunk. We measured gravimetric water content, conductivity, pH, extractable phosphorus (Mehlich‐III), total phosphorus, organic carbon, and total nitrogen on seven air‐dried soil samples to characterize soil properties at the site (Table 1). Overall, soils were sandy and relatively low in available water and nutrients. Roots were surface‐cleaned with 70% ethanol, rinsed three times with deionized water, and then cut in 1–5 mm long fragments. Samples of roots and approximately 15 mL of the soil samples were frozen at −4°C within 6 h of sampling for subsequent molecular analysis.

**Table 1:** Mean environmental variables and soil physicochemical properties for the whole sampling area. Values are means ± standard deviation (*n* = 32)

Elevation (m)	3.9 ± 0.7
pH	4.9 ± 0.3
Water content (%w)	7.7 ± 0.6
Organic horizons thickness (cm)	2.7 ± 0.3
Total nitrogen (g/kg)	1.16 ± 0.04
Organic carbon (g/kg)	7.1 ± 1.5
Bioavailable phosphorus (mg/kg)	8.7 ± 1.2
Total phosphorus (mg/kg)	42.6 ± 4.1

### Description of ectomycorrhizal fungal communities using 454 pyrosequencing

Roots fragments were disrupted using the TissueLyser (QIAGEN, Valencia, CA) with four 30‐sec cycles. We extracted total genomic DNA from 100 to 200 mg of root material using the NucleoMag 96 Plant DNA extraction kit (Macherey‐Nagel, D‐Mark Biosciences, Toronto, ON, Canada), and from 250 to 300 mg of soil material with the PowerSoil^™^ DNA Isolation Kit (MOBIO Laboratories, Carlsbad, CA) according to instructions by the manufacturer. The internal transcribed spacer (ITS) regions were then amplified using the ITS1F and ITS4 primers (White et al. 1990; Gardes & Bruns 1993). This region includes the two highly variable spacers ITS1 and ITS2, and the intercalary 5.8S gene. The directional GS FLX Titanium adaptors A and B (including a four‐base library key sequence) were attached at the 5′ end of the primers, and a unique 12‐bp Multiplex Identifier (MID) was added between the library key and the template‐specific sequence of the forward primer to allow sequences to be assigned to samples. We performed polymerase chain reaction (PCR) in triplicates using for each sample: 0.5 U of Qiagen Taq DNA Polymerase (Qiagen, Toronto, ON, Canada), 1× of the manufacturer's reaction buffer, 0.275 *μ*mol/L of each primer and dNTPs, a final concentration of 2.75 *μ*mol/L MgCl_2_, and 0.83 *μ*L each of 1% Tween‐20, DMSO, and BSA, as well as 2 *μ*L of diluted DNA (1:10) in a total volume of 20 *μ*L. The cycling conditions were 94°C for 5 min, followed by 32 cycles of 94°C for 45 sec, 55°C for 35 sec, and 72°C for 1 min, and a final elongation of 72 °C for 7 min. Triplicates were pooled, then purified with the NucleoMag 96 PCR clean‐up kit (Macherey‐Nagel; D‐Mark Biosciences, Toronto, ON, Canada), and quantified with the Qubit^®^ 2.0 Fluorometer (Life Technologies, Burlington, ON, Canada). An equal amount of amplified DNA from each sample was combined into a single pool and sent for pyrosequencing using Roche 454 GS FLX+ chemistry at the Genome Québec Innovation Center (McGill University, Montréal, QC, Canada).

We extracted and grouped *fasta* and *qual* files from the *sff* files provided by the sequencer using Mothur v.1.29.2 (Schloss et al. 2009) and then imported sequences into Qiime (Caporaso et al. 2010) for quality filtering and reassignment to samples. Low‐quality ends were trimmed using a minimum quality score of 25 within a moving window of 50 bp. We excluded sequences shorter than 200 bp, longer than 1000 bp, with more than two ambiguities (Ns), with homopolymers longer than 8 bp, or with two or more mismatches in the primer or barcode. Sequences were pruned to a fixed length (300 bp) with Mothur in order to avoid the partial coverage problem, which can result in erroneous or low‐quality consensus sequences (Edgar 2014). Chimera control and sequence clustering was done using Usearch v7.0 (Edgar 2010). Operational taxonomic units (OTUs) were determined at a similarity level of 97% and reads were then mapped back into an OTU table. All global singletons (i.e., OTUs represented by only one read in the whole data set) were eliminated to avoid any artifacts that could be attributed to sequencing errors (Tedersoo et al. 2010) and to improve the accuracy of diversity estimates (Ihrmark et al. 2012). Taxonomy was assigned to each OTU consensus sequence using the UNITE database (Kõljalg et al. 2013) in Mothur, which provides a naïve Bayes classifier with a minimum bootstrap value of 60%. Raw sequence data were deposited in the NCBI Sequence Read Archive and are available under the project number PRJNA286207. Identified OTUs were manually screened for potential EM interactions based on the more recent list of EM fungal taxa (Tedersoo and Smith 2013) and retained for further analysis. Finally, using the UNITE database, we also attributed an exploration type (cord‐forming or simple mycelia) as described by Agerer (2001) to each EM OTU.

### Fungal diversity assessment

All statistical analyses were conducted in R v.3.0.2 (R Foundation for Statistical Computing; available at http://www.R-project.org), unless indicated otherwise. In order to evaluate the adequacy of sampling and sequencing depth, and to allow for comparison of richness among samples, a rarefaction analysis was performed using the “iNEXT” package (Hsieh et al. 2013). Total OTU richness was evaluated with the Chao estimator (Chao 1984) and sample coverage was computed as suggested by Chao and Jost (2012). We also used the approach of rarefying each sample to an equal number of sequences and computed coverage again. We rarefied at the level of 100 reads, and five samples (two roots and three bulk soil) were discarded from the analysis because they were represented by <100 sequences. We conducted ANOVA and Tukey HSD post hoc tests using the “aov” and “TukeyHSD” functions of the “stats” package to identify significant differences among host tree species in terms of EM OTUs richness. We also assessed the taxonomical composition of roots and soil EM communities by computing relative read abundance and OTU richness of major fungal orders.

To explore the patterns of fungal OTU composition among samples, we used principal coordinate analysis (PCoA). The Hellinger distance was computed using the “decostand” and “vegdist” function in “vegan”. This distance metric emphasizes differences in relative rather than raw abundances (Ramette 2007; Anderson et al. 2011), which was deemed appropriate in this case because the number of reads is not a direct measure of OTU abundance in the environment. For completeness, we also used the Sorensen distance, which is completely unweighted (i.e., relying on presence–absence data). Permutational multivariate analysis of variance was performed with the “adonis” function in “vegan” with 9999 permutations to evaluate the statistical significance of differences in OTU community structure among host species and sample type (i.e., roots vs soil). When a main term was significant, we performed post hoc pairwise comparisons and corrected *P*‐values for multiple comparisons with the Holm correction, using the “p.adjust” function in the “stats” package.

### Quantification of network structure

A quantitative interaction matrix was constructed by computing the frequency (i.e., number of occurrences) of each EM OTU across the eight replicates for the four host species for both roots and bulk soil samples. To avoid describing patterns that are caused by very low‐frequency taxa, we only considered OTUs that were present in at least 10% of samples for the analysis. We computed two community‐level properties that are widely applied in the analysis of bipartite ecological networks (Fortuna et al. 2010; Deng et al. 2012): nestedness and modularity. Nestedness gives a measure of the degree of hierarchy in the organization of the interactions. High nestedness occurs when the most specialized species of one class interact mainly with the generalist species of the other class (Bascompte et al. 2003). Nestedness was measured as WNODF (Weighted Nestedness metric based on Overlap and Decreasing Fill), which is a quantitative adaptation the NODF index (Almeida‐Neto and Ulrich 2011). This index, which ranges from 0 to 100, was computed using the “FALCON” package (Beckett et al. 2014).

Modularity is a network property evaluating the presence of modules, that is, subgroups of closely connected species. A highly modular network would display a large number of modules and/or very well‐defined and secluded modules. Weighted modularity was assessed using the QuanBiMo algorithm recently developed by Dormann and Strauss (2014). This algorithm uses a hierarchical random graph approach (Clauset et al. 2008) which transposes the network into a dendrogram and randomly swaps branches to find the optimal division into modules and maximize Q. This approach is implemented in the “bipartite” package and available through the “computeModules” function. We used 10^7^ steps (or swaps) after which the run was terminated in the absence of further improvement. The degree *Q* of modularity, the number of detected modules, and the affiliation of species to modules were recorded from the optimal run. Significance of both nestedness and modularity was tested by comparing observed values to those of 1000 permuted matrices generated from a conservative null model that preserves row and column sums (Beckett et al. 2014).

Finally, we computed the paired difference index (PDI) with the “getspe” function of the “ESM” library in order to determine the degree of specificity of the different hosts and EM fungal OTUs. PDI is a robust specialization index that relies on continuous quantitative data, that is, on the strength of links between species, to classify species as generalists or specialists (Poisot et al. 2012). We also used the indicator species analysis to identify indicator OTUs of each host species, which is available in the “indicspecies” package in R (Cáceres and Legendre 2009). We conducted this analysis on presence–absence data. The indicator values were group‐equalized and their statistical significance was tested by a randomization procedure with 999 permutations. The “visweb” function of the “bipartite” package was used to produce a visual representation of EM OTUs occurrence across replicates for each host species. We used Cytoscape (Shannon et al. 2003) for visualizing our bulk soil and roots species interaction networks with the edge‐weighted spring embedded layout and edge representation weighted by their betweenness.

## Results

### Patterns in EM fungal richness

454 sequencing of the ITS regions and quality filtering yielded a total of 190,600 sequences which clustered into 1613 fungal OTUs, excluding chimeras and singletons. We retained 200 of these OTUs identified as EM fungi for further analysis (Tedersoo and Smith 2013); these were represented by 34,192 sequences, corresponding to approximately 18% of total reads (described in Table S1). One hundred and sixty‐eight of these OTUs were associated with roots samples and 185 with bulk soil samples. Rarefaction analysis indicated that our sequencing depth was adequate: All curves reached or were close to reaching an asymptote (Figure S1) and all samples had a Good's coverage value close to 1 (Table S2). Rarefaction analysis also showed that we accomplished sufficient sampling effort, that is, detection of over 50% of fungal taxa (Bahram et al. 2014). Based on the Chao estimator of total richness, we detected 65.28% of taxa in soil communities and 71.92% in root communities in average. We recorded 16 OTUs on average in each root sample and of 22 OTUs in each bulk soil sample.

**Figure 1:**
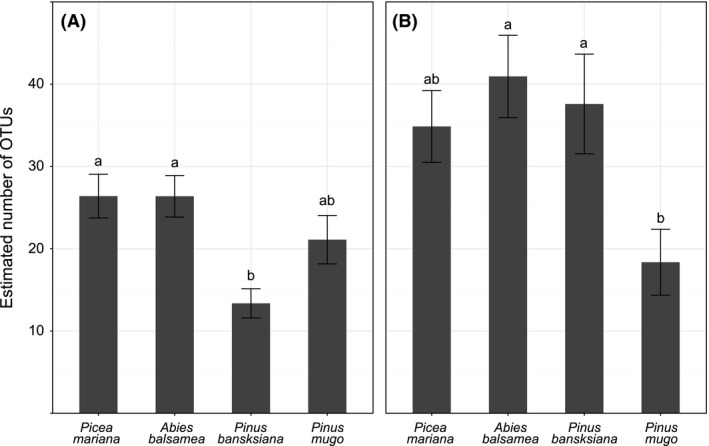
Estimated total OTU richness across the different host species for roots (A) and soil (B) samples. Total richness was evaluated with the Chao estimator. Different letters indicate significant differences among hosts (*P ≤ *0.05).

There were no significant differences in rarefied OTU richness among hosts (Table S2). However, there were some significant differences in Chao estimator values for total OTU richness (Fig. 1). In particular, *Picea mariana* and *A. balsamea* supported a higher root‐associated rarefied OTU richness than that found in *Pinus banksiana*. Also, as much as 50% lower average richness (both observed and total) was observed in soils associated with *Pinus mugo* compared to some other host species. Specifically, bulk soil EM fungal richness in *A. balsamea* and *Pinus banksiana* was significantly greater than that observed in *Pinus mugo* (Fig. 1).

### Community structure

**Figure 2:**
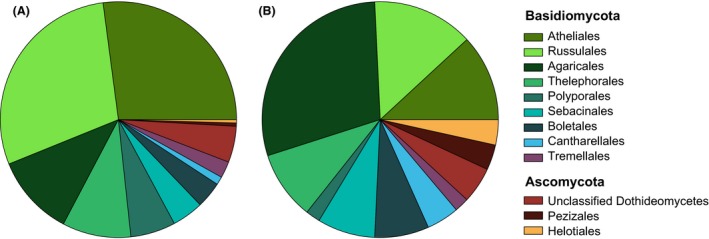
Frequency distribution of EM fungal taxa in terms of (A) number of reads and (B) number of OTUs.

Ectomycorrhizal OTUs belonged to 51 different genera, 31 families, 14 orders and five classes (Agaricomycetes, Pezizomycetes, Leotiomycetes, Dothideomycetes, and Sordariomycetes; Fig. 2). Agaricomycetes were the dominant class in terms of both relative sequence abundance and number of OTUs. Two families (Russulaceae and Atheliaceae) together accounted for over 50% of total sequences and contributed significantly to overall OTU richness, with 23 and 22 OTUs in each group, respectively (Table S3). Russulaceae and Atheliaceae, together with Cortinariaceae and Sebacinaceae, represented half of the total EM richness observed (100 OTUs). There was considerable variation in the composition of root and bulk soil associated EM fungal communities within replicate individuals of the same tree species, and only a few significant differences among host species were detected, depending on the distance or dissimilarity metric used (Figure S2). Analyses based on the Hellinger distance revealed that *Picea mariana* supported soil communities that were significantly different from those found in *Pinus banksiana* (*P *=* *0.009) and *Pinus mugo* (*P *=* *0.0295), while the analysis based on the Sorensen dissimilarity showed a significant difference between the root‐associated communities of *Picea mariana* and those of *Pinus banksiana* (*P *=* *0.0390).

### Architecture of belowground plant host–EM fungi interaction networks

Our analysis revealed significant antinestedness in the tree host–EM fungal interaction network. Indeed, we calculated a weighted NODF of 25.28 for roots and of 29.40 for bulk soil network, which corresponded to a significantly lower (corrected *P*‐value ≤ 0.05) level of nestedness than that expected under a null model (roots, mean WNODF: 33.79, standard deviation: 2.18, *Z*‐score: −3.90; soils, mean WNODF: 34.87, standard deviation: 1.91, *Z*‐score: −2.86). On the other hand, modularity values for both roots and bulk soil were relatively low and did not differ significantly from the null expectation. We recorded modularity levels of 0.1914 and 0.1521 against expected means of 20.05 and 0.1788 (standard deviations: 0.0169 and 0.0166; *z*‐scores: −0.5422 and 1.6191 for roots and bulk soil network, respectively (Figure S3).

**Figure 3:**
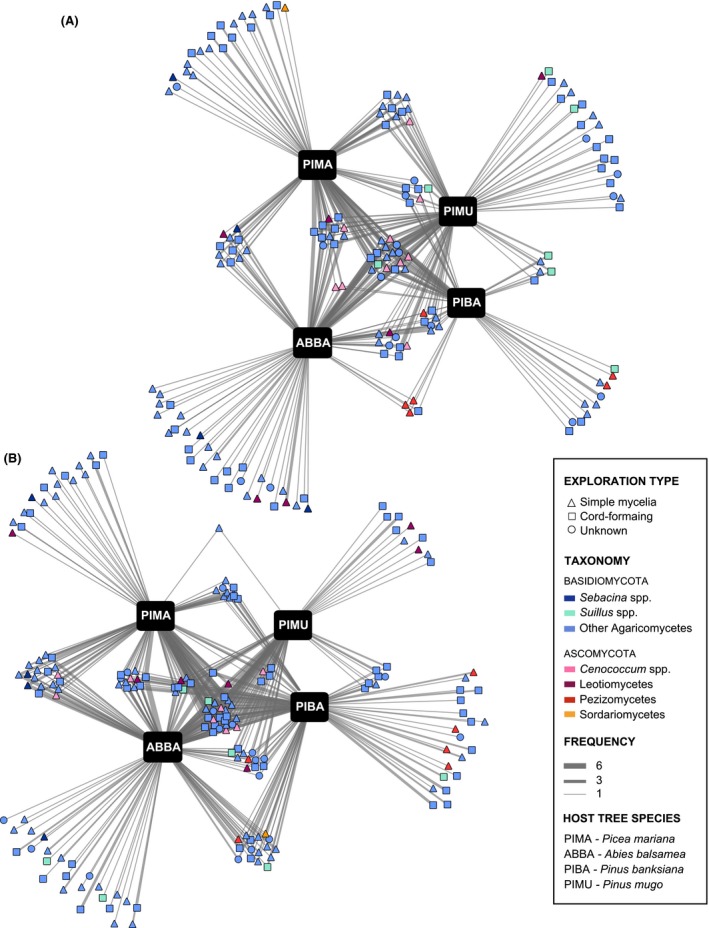
(A) Roots and (B) bulk soil EM interaction networks. The four hosts are represented by black boxes; EM fungal OTUs are colored according to their taxonomy and their shape indicates exploration type. Edges (i.e., links between EM fungal OTUs and host trees) width is proportional to the frequency of observation.

Despite the fact that ordination analysis highlighted only minor differences in EM community composition among hosts, a large proportion of OTUs was associated with only one host species (51.1% for roots and 39.5% for bulk soil) (Fig. 3). There was also a large number of OTUs that were associated with three of the four host species (14.0% and 16.8%) and with all hosts (10.4% and 13.5%). We recorded slightly more OTUs associated with a simple mycelia exploration strategy (94 OTUs in the bulk soil samples and 86 in roots samples) than cord‐forming fungi (73 in bulk soil and 65 in roots). Degree distribution did not differ between the two exploration types (cord‐forming and simple mycelia) as revealed by a chi‐square test (*χ* ^2^ = 7.2993, df = 6, *P*‐value = 0.2941) (Figure S4).

Our data revealed some phylogenetic patterns in host–EM interactions. In both roots and bulk soil samples, EM OTUs belonged predominantly to the Agaricomycetes. In addition, all observed Pezizomycetes were associated with *Pinus banksiana*, whereas some *Sebacina* were strictly associated with *Picea mariana* and *Abies balsamea*. Operational taxonomical units belonging to the Dothideomycetes always interacted with at least two different host species and often three or four, and thus could be considered as generalists. We recorded only one OTU from the Sordariomycetes.

### Plant–EM fungal associations

**Figure 4:**
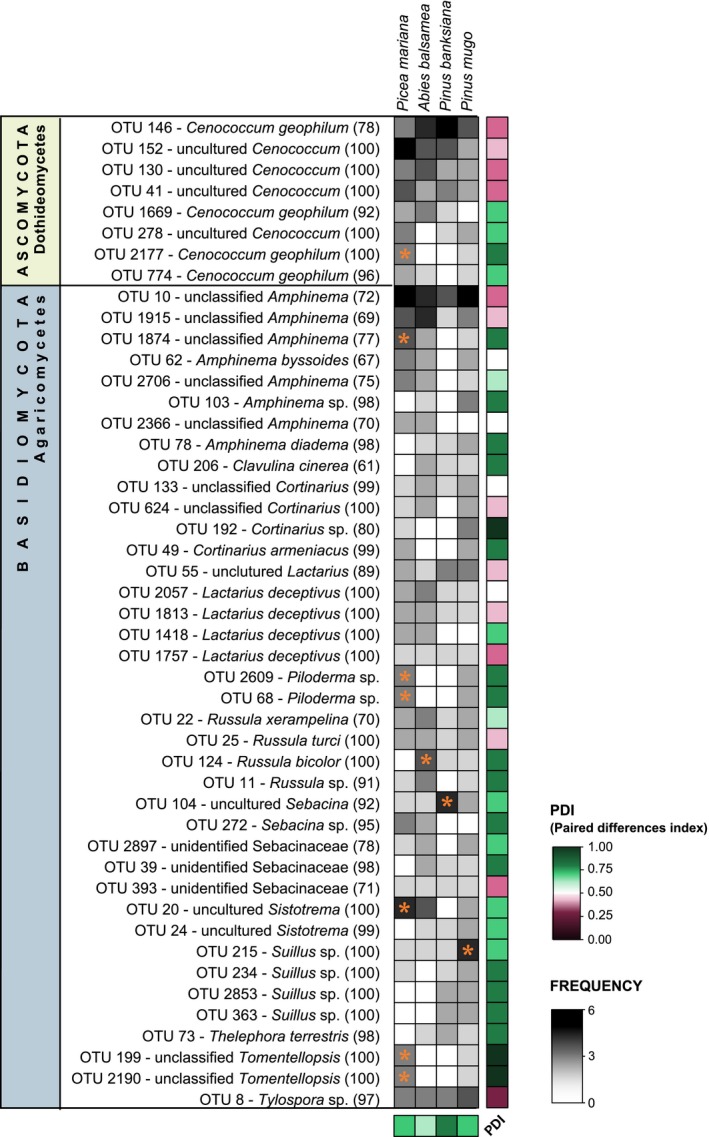
Association specificity of EM fungal OTUs with a frequency of at least 0.1 in tree roots showing frequency across replicates for each host species and PDI values. PDI is a specialization index ranging from 0 (generalist) to 1 (specialist). Orange stars indicate significant (*P *≤* *0.05) associations (i.e., OTUs having a significant indicator value of at least 0.5). Species attributed to each OTUs correspond to identity assigned using the UNITE database and numbers in parenthesis are the bootstrap values.

Despite the low overall degree of host specialization among EM fungal OTUs revealed by network analyses, we still observed a number of specialized EM fungal OTUs (Fig. 4). From the 47 OTUs that occurred in at least 10% of samples (i.e., frequency > 0.1), 29 had a PDI above 0.5 and could therefore be considered as specialists. By contrast, only 13 OTUs had a PDI below 0.5 and could be defined as generalists. *Tylospora* sp., three *Cenococcum* OTUs, an unclassified *Amphinema*, and a *Lactarius deceptivus* were the generalist OTUs with the lowest PDI, as well as an unidentified Sebacinaceae. On the other hand, some OTUs were mostly restricted to particular hosts. For example, indicator species analysis designated *Piloderma* sp., *Tomentellopsis* sp., a *Cenococcum geophilum*, an unclassified *Amphinema,* and an uncultured *Sistoderma* as privileged partners of *Picea mariana* (Fig. 4). Operational taxonomy unit 124 (*Russula bicolor*) was identified as a specialist of *A. balsamea*, OTU 104 (uncultured *Sebacina*) of *Pinus banksiana*, and OTU 215 (*Suillus* sp.) of *Pinus mugo*. The PDI of all host trees was above 0.5, which classifies them as specialists and indicates that their roots support distinct EM fungal communities.

## Discussion

Using an ITS‐targeted pyrosequencing approach, we were able to describe EM fungal communities among four co‐occurring tree hosts in a coastal dune boreal forest. Network analysis of plant–EM fungal interactions revealed an antinested pattern, but we could not detect significant modules of closely interacting species. Furthermore, we did not observe clear patterns of variations in OTU richness nor community composition among host species. Despite the relative low level of host preference observed, a number of less frequent OTUs nonetheless appeared to show some degree of specialization to a given host. The computation of PDI index as well as indicator species analysis allowed us to identify some specialist OTUs displaying specialized patterns of association. Generalists – that is, OTUs associated with several or all hosts – still represented a large proportion of the EM communities and might be important for community resilience as they improve connectivity in the network and enhance its stability. Overall, our findings point to relatively low levels of specialization of EM associations, contrary to our initial expectation of community distinctiveness and host preference.

Despite the generally low level of specialization of EM association observed overall, OTUs interacting with only one host species represented an important proportion of recorded EM richness (51.1% of OTUs for roots and 39.5% for bulk soil). *Abies balsamea* supported the higher number of rare OTUs (OTUs encountered in only one root or soil sample) with 20 in soil samples and 28 in root samples. The number of rare OTUs per host species ranged between nine and 28. Even if it is difficult to discriminate OTUs that are preferentially associated with a given host from those infrequent species with undetected occurrences (Bahram et al. 2014), we are confident that most OTUs we identified as specialists are so, because rarefaction analysis indicated adequate sequencing depth. In addition, we met the 50% threshold of sampling effort, that is, detection of at least 50% of taxon for the community, as recommended by Bahram et al. (2014). Operational taxonomic units of the *Sebacina* genus appeared to specialize on *Picea mariana* and *Abies balsamea*. Interestingly, *Picea*‐*Sebacina* associations were reported before (Warcup 1988; Baier et al. 2006). *Suillus* and other members of the sulloid group are known to display narrow association patterns, most species specializing on a single host genus, and are known as exclusive symbionts of Pinaceae (Cairney and Chambers 1999; Smith and Read 2008). In this study, seven different OTUs were identified as members of the *Suillus* genus which seemed to be found more frequently in association with the two *Pinus* – especially OTU 215, identified as a significant indicator of *Pinus mugo*. Association with specialists such as *Suillus* might be advantageous for the host tree if it reduces the risks of resources diversion to other competing tree species through the common mycelial network (Molina et al. 1992; Smith and Read 2008). Such functional outcomes of specialist plant–fungal interactions deserve to be further explored.

Despite the presence of these rare EM fungal OTUs, only a few minor significant differences in richness and community composition were detected. Overall, our results therefore indicate a relatively low level of host preference. We recorded a large proportion of fungal OTUs interacting with three or four different hosts. Even if no OTU was found in all roots or soil samples (the most widespread OTU, an *Amphinema* species, was present in around 70% of all samples), these taxa can interact with diverse host species and are potential generalists. In our network analysis, all OTUs from the *Cenococcum* genus were connected to several hosts and therefore could be seen as generalists. This genus is known to be a taxon with a very wide distribution and *C. geophilum* – one of the most widespread EM fungal species in the environment with an exceptionally wide habitat range – is considered as a “super‐generalist” (Cairney and Chambers 1999; Smith and Read 2008). It is also known to act as a pioneer species and invade newly formed soils such as glacial moraines, volcanic ash, and sand dunes during primary succession (Cairney and Chambers 1999). In our study, four OTUs of the genus *Cenococcum* were classified as generalists (OTUs 146, 152, 130 and 41). The functions and benefits of these taxa as a cosmopolitan EM fungi are, however, still debated (Smith and Read 2008). Generalist taxa might contribute to network resilience by increasing connectivity within the network which enhances its stability, whereas specialists enhance diversity (Bascompte and Jordano 2007). Both categories contribute importantly to network structure.

Network analyses have gained recent interest to explore the structure of mutualistic interaction networks (Bascompte et al. 2003; Bascompte and Jordano 2007; Bastolla et al. 2009), including mycorrhizal interactions (Bahram et al. 2014; e.g., Chagnon et al. 2012; Jacquemyn et al. 2015). While mutualistic plant–pollinator networks generally display a nested structure, which minimizes interspecific competition and enhances diversity (Bastolla et al. 2009), most studies on ectomycorrhizal networks have not detected such a nested pattern (Bahram et al. 2014). As such, the antinested patterns we observed in both root‐associated and bulk soil interaction networks are consistent with the findings of Bahram et al. (2014), where the authors found antinested networks in half of the data sets analyzed. This pattern could have suggested the presence of subgroups of closely interacting species, that is, modules. However, contrary to our hypothesis, we did not find significant modularity in EM interaction networks. The *Q* values of modularity that we recorded were much lower – with one exception – than the modularity levels recorded by Bahram et al. (2014) and were not significantly different from the null expectations. The absence of a positive nested pattern could be attributed to the fact that we sampled only four host species, which could be insufficient to statistically detect nestedness (Bascompte et al. 2003). Larger networks were generally found to be more nested than smaller networks (Bascompte et al. 2003), suggesting that our estimates of nestedness might have been conservative. Low and nonsignificant modularity, on the other hand, indicates the absence of specialized associations. Modularity measures are known to be sensitive to the total number of achieved links in a network, that is, the existence of specialized interactions could be obscured by the presence of a host with a high number of links. As such, Bahram et al. (2014) were able to detect higher and significant modularity by achieving equalized random sampling (i.e., randomly selecting the same number of EM OTUs for each host). Still, in the present study, the absence of modules is consistent with the observation of a large proportion of fungal OTUs associating with three or four different hosts. Moreover, OTUs interacting preferentially with one host species were infrequent – as discussed above, most of them were detected in a single sample – resulting in a nonmodular network structure.

In recent studies, host identity has often been identified as the strongest predictor of EM fungal community composition and structure (e.g., Ishida et al. 2007; Morris et al. 2009; Tedersoo et al. 2013), and has even been proposed as the main determinant of EM fungal community structure (Murata et al. 2013). Still, the extent of this host effect remains incompletely understood, mainly because of the difficulty to unravel the complex interactions between host plants, microbial communities, and soil and environmental properties (Aponte et al. 2010; Peay et al. 2015). A major challenge in the interpretation of field studies results is the confounding effect of environmental covariation affecting both tree hosts and EM fungi. In this study, we sampled a relatively small section of the dune system with relatively homogenous environmental, climatic, and edaphic conditions, thus allowing us to examine EM fungal community structure and network architecture with minimal variations of the abiotic environment. Still, we did not observe clear effects of host identity on EM fungal richness or community composition. We recorded a total of 200 EM OTUs and the richness levels we observed in samples ranged from 4 to 46 and estimated total richness from 4 to 63, which is high but within the same range as other EM studies conducted in different ecosystems (e.g., Ishida et al. 2007; Aponte et al. 2010; Murata et al. 2013; Tedersoo et al. 2013; Peay et al. 2015). Overall, our results indicate that while total EM fungal diversity was high, all hosts mostly showed similar levels of EM fungal diversity in their roots. Ordination analysis did not reveal clear segregation among host species in terms of EM fungal community structure either. The level of intrahost variability was high and we observed only few significant differences among EM communities associated with the different hosts.

One potential explanation of the low variation in community composition among host species would be that the four tree species all belong to the same family (Pinaceae), and as such, plant–EM fungal interactions might be well conserved at the family level. Taxonomic relatedness was indeed found to be one of the main factor governing host effect among Salicaceae trees (Tedersoo et al. 2013; Bell et al. 2014). Multihost studies in temperate mixed forests also revealed positive correlations between host taxonomic distance and the distinctiveness of the EM communities they support (Ishida et al. 2007; Morris et al. 2009; Murata et al. 2013). Peay et al. (2015) also studied closely related hosts (13 genera of Dipterocarpaceae) and observed weak differentiation of EM communities. This study showed that edaphic specialization (i.e., similar reaction of microbes and plants to edaphic conditions) accounted for the covariation observed among host taxonomy and EM community composition, not host specialization itself. Conversely, some plant families or genera such as *Alnus* are also known to display highly specific patterns of interactions, even at a regional scale (Roy et al. 2013). Other studies also found that host phylogeny explained important proportions of variation in EM communities associated with members of the Salicaceae (Tedersoo et al. 2013; Bell et al. 2014). Moreover, the few significant differences that we detected in both richness and community structure separated *Picea mariana* from the two *Pinus* species, reinforcing the idea that taxonomic relatedness is a key factor governing host effect and explaining variations in EM community composition. It is possible that genotypic variations of closely related host species trigger changes in EM fungal communities (Sthultz et al. 2009) if associated phenotypes are ecologically divergent (Peay et al. 2015), which might not be the case in our study. In the particular case of Pinaceae, there are several earlier reports of an important overlap in EM community composition among different tree species suggesting the predominance of multiple host fungi (e.g., Horton and Bruns 1998; Kranabetter et al. 1999; Cullings et al. 2000). For example, Horton et al. (2005) found as much as 95% of EM species in common between western hemlock seedlings and co‐occurring Douglas fir. This could also explain the low and nonsignificant modularity we observed.

In conclusion, our study revealed no strong effect of host identity on EM richness and community composition associated with four co‐occurring Pinaceae. The examination of network structural properties suggests a relatively low level of host specialization in these EM interactions. The lack of differences in EM fungal richness and community structure among hosts, as well as the absence of specialized subgroups of interactions (modules), could be attributed to the taxonomic relatedness and ecological similarity of our four host tree species, which all belong to the same family (Pinaceae). Therefore, important effects of host identity might operate at a higher taxonomic level. Low specificity might be advantageous for host trees by increasing their chance to find suitable EM partners. This could be ecologically important, especially in a nutrient‐poor environment such as sand dunes where trees rely strongly on EM fungi for nutrient uptake. We observed a high level of EM diversity (200 OTUs) despite the absence of fungal community differentiation among the four co‐occurring hosts, raising the question of how such high fungal diversity is maintained if hosts have similar EM fungal community composition. Future studies should attempt to determine which mechanisms limit competitive exclusion among EM fungal species and allow the coexistence of a large number of EM fungal species in this habitat. Our study, as a first analysis of EM interactions in a coastal dune forest, provides further insights about the architecture of tree root–EM fungal species interaction networks and also raises some questions about the mechanisms promoting EM fungal species coexistence.

## Data Accessibility

The raw.sff files from 454 sequencing have been deposited in the Sequence Read Archive with Accession no. SRP059280. Available at: http://www.ncbi.nlm.nih.gov/Traces/sra/?study=SRP059280.

## Conflict of Interest

None declared.

## Supporting information


**Table S1.** List of EM fungal OTUs detected in roots or bulk soil of the four host species.
**Table S2.** Mean and standard error of number of reads, richness and coverage of EM fungal OTUs with ANOVA testing.
**Table S3.** Total relative abundance of the different EM fungal families encountered in roots and bulk soil samples in terms of number of OTUs and number of reads.
**Figure S1.** Rarefaction curves of EM fungal OTUs in roots (A–D) and soil (E–H) samples against the number of 454 reads excluding singletons for *Picea mariana* (A, E), *Abies balsamea* (B, F), *Pinus banksiana* (C, G), and *Pinus mugo* (D, H).
**Figure S2.** Principal coordinate analysis (PCoA) of roots (A‐B) and bulk soil (C–D) associated EM fungal community based the Hellinger distance (A and C) and the Sorensen (B and D) dissimilarity.
**Figure S3.** Modularity and nestedness of roots and bulk soil data in relation to the 1000 matrices generated with a null model preserving rows and columns sums.
**Figure S4.** Degree distribution of EM fungal OTUs in function of exploration type.
