# Supplementary material for: High richness of ectomycorrhizal fungi and low host specificity in a coastal sand dune ecosystem revealed by network analysis
Source: Ecol Evol. 2015 Dec 29;6(1):349–62. doi: 10.1002/ece3.1881 (PMC4716518; doi:10.1002/ece3.1881)
Supplement: Supplementary file 1 — Table S1. List of EM fungal OTUs detected in roots or bulk soil of the four host species. Table S2. Mean and standard error of number of reads, richness and coverage of EM fungal OTUs with ANOVA testing. Table S3. Total relative abundance of the different EM fungal families encountered in roots and bulk soil samples in terms of number of OTUs and number of reads. Figure S1. Rarefaction curves of EM fungal OTUs in roots (A–D) and soil (E–H) samples against the number of 454 reads excluding singletons for Picea mariana (A, E), Abies balsamea (B, F), Pinus banksiana (C, G), and Pinus mugo (D, H). Figure S2. Principal coordinate analysis (PCoA) of roots (A‐B) and bulk soil (C–D) associated EM fungal community based the Hellinger distance (A and C) and the Sorensen (B and D) dissimilarity. Figure S3. Modularity and nestedness of roots and bulk soil data in relation to the 1000 matrices generated with a null model preserving rows and columns sums. Figure S4. Degree distribution of EM fungal OTUs in function of exploration type. [file ECE3-6-349-s001.docx]

**Supporting information**

**Table S1** List of EM fungal OTUs detected in roots or bulk soil of the four host species. The number in parenthesis correspond to the bootstrap value for the species attribution using the UNITE database

|  |  | Family | | Genus | UNITE species attribution | | Total number of reads | Total number of occurrence | Occurrence in roots samples | | | | | | | Occurrence in bulk soil samples | | | | | |
| --- | --- | --- | --- | --- | --- | --- | --- | --- | --- | --- | --- | --- | --- | --- | --- | --- | --- | --- | --- | --- | --- |
|  |  |  |  |  |  |  |  |  | PIMA | ABBA | | PIBA | | PIMU | | PIMA | | ABBA | | PIBA | PIMU |
| Basidiomycota - Agaricomycetes | | | |  |  | |  |  |  |  | |  | |  | |  | |  | |  |  |
| OTU | 230 | Albatrellaceae | | *Leucophleps* | Leucophleps_spinispora (95) | | 163 | 6 | 0 | 1 | | 0 | | 0 | | 0 | | 3 | | 2 | 0 |
| OTU | 913 | Albatrellaceae | | *Leucophleps* | Leucophleps_spinispora (90) | | 35 | 3 | 0 | 1 | | 0 | | 0 | | 0 | | 1 | | 1 | 0 |
| OTU | 1948 | Albatrellaceae | | *Leucophleps* | Leucophleps_spinispora (99) | | 4 | 2 | 0 | 0 | | 0 | | 0 | | 0 | | 1 | | 1 | 0 |
| OTU | 3061 | Albatrellaceae | | *Leucophleps* | Leucophleps_spinispora (99) | | 8 | 2 | 0 | 0 | | 0 | | 0 | | 0 | | 1 | | 1 | 0 |
| OTU | 3152 | Albatrellaceae | | *Leucophleps* | Leucophleps_spinispora (95) | | 75 | 4 | 0 | 1 | | 0 | | 0 | | 0 | | 2 | | 1 | 0 |
| OTU | 204 | Amanitaceae | | *Amanita* | unculturedfungus (100) | | 158 | 4 | 0 | 0 | | 1 | | 1 | | 1 | | 0 | | 0 | 1 |
| OTU | 237 | Amanitaceae | | *Amanita* | unclassified | | 142 | 4 | 0 | 1 | | 0 | | 0 | | 1 | | 1 | | 1 | 0 |
| OTU | 335 | Amanitaceae | | *Amanita* | unculturedfungus (100) | | 70 | 6 | 2 | 1 | | 0 | | 0 | | 2 | | 1 | | 0 | 0 |
| OTU | 62 | Atheliaceae | | *Amphinema* | Amphinema_byssoides (67) | | 1218 | 21 | 3 | 2 | | 0 | | 2 | | 5 | | 6 | | 3 | 0 |
| OTU | 78 | Atheliaceae | | *Amphinema* | Amphinema_diadema (98) | | 558 | 16 | 0 | 1 | | 1 | | 2 | | 1 | | 4 | | 4 | 3 |
| OTU | 94 | Atheliaceae | | *Amphinema* | Amphinema_sp_6_UK_2011 (100) | | 231 | 6 | 0 | 1 | | 1 | | 0 | | 1 | | 2 | | 1 | 0 |
| OTU | 483 | Atheliaceae | | *Amphinema* | unculturedfungus (99) | | 50 | 6 | 0 | 0 | | 1 | | 0 | | 1 | | 2 | | 2 | 0 |
| OTU | 1357 | Atheliaceae | | *Amphinema* | Amphinema_byssoides (100) | | 27 | 4 | 0 | 0 | | 0 | | 0 | | 1 | | 2 | | 1 | 0 |
| OTU | 1874 | Atheliaceae | | *Amphinema* | unclassified | | 15 | 8 | 4 | 2 | | 0 | | 1 | | 0 | | 0 | | 1 | 0 |
| OTU | 2051 | Atheliaceae | | *Amphinema* | Amphinema_byssoides (84) | | 29 | 3 | 0 | 0 | | 0 | | 0 | | 0 | | 3 | | 0 | 0 |
| OTU | 2366 | Atheliaceae | | *Amphinema* | unclassified | | 11 | 9 | 2 | 2 | | 0 | | 0 | | 1 | | 0 | | 1 | 3 |
| OTU | 2706 | Atheliaceae | | *Amphinema* | unclassified | | 58 | 12 | 3 | 2 | | 0 | | 1 | | 1 | | 1 | | 2 | 2 |
| OTU | 10 | Atheliaceae | | *Amphinema* | unclassified | | 2623 | 46 | 6 | 5 | | 4 | | 6 | | 7 | | 8 | | 5 | 5 |
| OTU | 103 | Atheliaceae | | *Amphinema* | Amphinema_sp_6_UK_2011 (98) | | 333 | 14 | 0 | 1 | | 0 | | 3 | | 2 | | 1 | | 3 | 4 |
| OTU | 2200 | Atheliaceae | | *Amphinema* | Amphinema_sp_6_UK_2011 (98) | | 18 | 2 | 0 | 0 | | 0 | | 1 | | 0 | | 0 | | 0 | 1 |
| OTU | 1915 | Atheliaceae | | *Amphinema* | unclassified | | 782 | 31 | 4 | 5 | | 1 | | 3 | | 6 | | 5 | | 4 | 3 |
| OTU | 68 | Atheliaceae | | *Piloderma* | Piloderma_sp_9_RT_2012 (75) | | 416 | 10 | 3 | 0 | | 0 | | 2 | | 4 | | 0 | | 1 | 0 |
| OTU | 117 | Atheliaceae | | *Piloderma* | Piloderma_olivaceum (100) | | 30 | 3 | 0 | 0 | | 0 | | 0 | | 2 | | 1 | | 0 | 0 |
| OTU | 123 | Atheliaceae | | *Piloderma* | Piloderma_sp_10_RT_2012 (100) | | 380 | 4 | 1 | 1 | | 0 | | 0 | | 1 | | 1 | | 0 | 0 |
| OTU | 179 | Atheliaceae | | *Piloderma* | Piloderma_olivaceum (96) | | 1 | 1 | 0 | 0 | | 0 | | 0 | | 0 | | 1 | | 0 | 0 |
| OTU | 2609 | Atheliaceae | | *Piloderma* | Piloderma_sp_9_RT_2012 (85) | | 84 | 9 | 3 | 0 | | 0 | | 2 | | 3 | | 0 | | 1 | 0 |
| OTU | 8 | Atheliaceae | | *Tylospora* | Tylospora_sp_4_RT_2012 (97) | | 2055 | 33 | 3 | 3 | | 3 | | 4 | | 6 | | 7 | | 3 | 4 |
| OTU | 109 | Atheliaceae | | *Tylospora* | Tylospora_fibrillosa (100) | | 248 | 6 | 0 | 0 | | 1 | | 1 | | 0 | | 2 | | 2 | 0 |
| OTU | 2960 | Atheliaceae | | *Tylospora* | Tylospora_sp_4_RT_2012 (94) | | 2 | 2 | 0 | 0 | | 0 | | 1 | | 1 | | 0 | | 0 | 0 |
| OTU | 2897 | Atheliaceae | | *unidentified* | unculturedPiloderma (78) | | 88 | 6 | 1 | 2 | | 0 | | 2 | | 0 | | 1 | | 0 | 0 |
| OTU | 66 | Bankeraceae | | *Boletopsis* | Boletopsis_sp. (94) | | 8 | 2 | 1 | 0 | | 0 | | 0 | | 0 | | 0 | | 1 | 0 |
| OTU | 2179 | Bankeraceae | | *Hydnellum* | Hydnellum_subsuccosum (100) | | 2 | 1 | 0 | 0 | | 0 | | 0 | | 1 | | 0 | | 0 | 0 |
| OTU | 1329 | Bankeraceae | | *Phellodon* | Phellodon_tomentosus (63) | | 3 | 2 | 0 | 1 | | 0 | | 0 | | 1 | | 0 | | 0 | 0 |
| OTU | 116 | Bankeraceae | | *Sarcodon* | Sarcodon_glaucopus (100) | | 214 | 7 | 1 | 1 | | 0 | | 0 | | 2 | | 3 | | 0 | 0 |
| OTU | 1127 | Bolbitiaceae | | *Alnicola* | Alnicola_sp_MG_2011b (88) | | 3 | 1 | 0 | 0 | | 0 | | 0 | | 0 | | 0 | | 1 | 0 |
| OTU | 107 | Bolbitiaceae | | *Hebeloma* | unclassified | | 341 | 4 | 0 | 1 | | 0 | | 1 | | 0 | | 1 | | 0 | 1 |
| OTU | 144 | Bolbitiaceae | | *Hebeloma* | Hebeloma_velutipes (100) | | 224 | 2 | 0 | 1 | | 0 | | 0 | | 0 | | 1 | | 0 | 0 |
| OTU | 842 | Bolbitiaceae | | *Hebeloma* | Hebeloma_sp_K06C5T156a (74) | | 34 | 2 | 0 | 1 | | 0 | | 1 | | 0 | | 0 | | 0 | 0 |
| OTU | 300 | Boletaceae | | *Boletus* | Xerocomus_badius (77) | | 53 | 8 | 2 | 0 | | 1 | | 0 | | 3 | | 0 | | 2 | 0 |
| OTU | 356 | Boletaceae | | *Boletus* | Boletus_sp_B334 (77) | | 121 | 8 | 1 | 0 | | 0 | | 1 | | 1 | | 1 | | 3 | 1 |
| OTU | 771 | Boletaceae | | *Boletus* | Boletus_edulis (100) | | 5 | 1 | 0 | 1 | | 0 | | 0 | | 0 | | 0 | | 0 | 0 |
| OTU | 1234 | Cantharellaceae | | *unidentified* | unculturedCantharellaceae (90) | | 4 | 1 | 0 | 0 | | 0 | | 0 | | 0 | | 1 | | 0 | 0 |
| OTU | 1917 | Cantharellaceae | | *unidentified* | unculturedCantharellaceae (61) | | 4 | 2 | 0 | 0 | | 0 | | 0 | | 0 | | 1 | | 1 | 0 |
| OTU | 833 | Cantharellaceae | | *unidentified* | unculturedCantharellaceae (76) | | 8 | 3 | 0 | 0 | | 0 | | 0 | | 0 | | 2 | | 1 | 0 |
| OTU | 1043 | Ceratobasidiaceae | | *Ceratobasidium* | Ceratobasidium_sp_257 (74) | | 1 | 1 | 0 | 0 | | 0 | | 1 | | 0 | | 0 | | 0 | 0 |
| OTU | 2965 | Ceratobasidiaceae | | *Ceratobasidium* | Ceratobasidium_sp_257 (71) | | 3 | 2 | 0 | 0 | | 0 | | 1 | | 0 | | 0 | | 1 | 0 |
| OTU | 206 | Clavulinaceae | | *Clavulina* | Clavulina_cinerea (61) | | 192 | 9 | 0 | 2 | | 1 | | 1 | | 2 | | 2 | | 1 | 0 |
| OTU | 449 | Clavulinaceae | | *Clavulina* | Clavulina_cinerea (62) | | 100 | 4 | 1 | 1 | | 0 | | 0 | | 1 | | 1 | | 0 | 0 |
| OTU | 460 | Clavulinaceae | | *Clavulina* | unculturedfungus (78) | | 27 | 6 | 2 | 0 | | 0 | | 0 | | 1 | | 2 | | 0 | 1 |
| OTU | 42 | Cortinariaceae | | *Cortinarius* | unclassified | | 96 | 4 | 0 | 0 | | 1 | | 1 | | 1 | | 0 | | 1 | 0 |
| OTU | 49 | Cortinariaceae | | *Cortinarius* | Cortinarius_armeniacus (99) | | 578 | 8 | 2 | 0 | | 0 | | 2 | | 2 | | 1 | | 0 | 1 |
| OTU | 133 | Cortinariaceae | | *Cortinarius* | unclassified | | 285 | 10 | 1 | 2 | | 1 | | 2 | | 2 | | 1 | | 0 | 1 |
| OTU | 135 | Cortinariaceae | | *Cortinarius* | unculturedfungus (100) | | 144 | 9 | 1 | 0 | | 0 | | 1 | | 3 | | 2 | | 1 | 1 |
| OTU | 167 | Cortinariaceae | | *Cortinarius* | Cortinarius_aureifolius (100) | | 234 | 1 | 0 | 0 | | 0 | | 1 | | 0 | | 0 | | 0 | 0 |
| OTU | 192 | Cortinariaceae | | *Cortinarius* | Cortinarius_sp. (80) | | 114 | 7 | 1 | 0 | | 0 | | 3 | | 0 | | 1 | | 1 | 1 |
| OTU | 198 | Cortinariaceae | | *Cortinarius* | Cortinarius_neofurvolaesus (99) | | 16 | 4 | 2 | 1 | | 0 | | 0 | | 0 | | 0 | | 1 | 0 |
| OTU | 211 | Cortinariaceae | | *Cortinarius* | unculturedfungus (88) | | 28 | 9 | 1 | 0 | | 1 | | 0 | | 2 | | 2 | | 2 | 1 |
| OTU | 228 | Cortinariaceae | | *Cortinarius* | Cortinarius_anomalus (98) | | 68 | 4 | 2 | 0 | | 0 | | 0 | | 0 | | 0 | | 2 | 0 |
| OTU | 261 | Cortinariaceae | | *Cortinarius* | Cortinarius_sp. (66) | | 52 | 3 | 0 | 1 | | 0 | | 0 | | 0 | | 1 | | 1 | 0 |
| OTU | 288 | Cortinariaceae | | *Cortinarius* | Cortinarius_casimiri (79) | | 70 | 9 | 0 | 1 | | 0 | | 1 | | 2 | | 3 | | 0 | 2 |
| OTU | 329 | Cortinariaceae | | *Cortinarius* | Cortinarius_sp_OSC_1064189 (92) | | 57 | 3 | 1 | 0 | | 0 | | 0 | | 1 | | 0 | | 1 | 0 |
| OTU | 369 | Cortinariaceae | | *Cortinarius* | unculturedfungus (74) | | 39 | 2 | 0 | 0 | | 0 | | 0 | | 0 | | 0 | | 1 | 1 |
| OTU | 397 | Cortinariaceae | | *Cortinarius* | unculturedfungus (74) | | 49 | 6 | 0 | 0 | | 0 | | 2 | | 0 | | 1 | | 3 | 0 |
| OTU | 407 | Cortinariaceae | | *Cortinarius* | Cortinarius_sp. (60) | | 21 | 2 | 0 | 1 | | 0 | | 0 | | 0 | | 1 | | 0 | 0 |
| OTU | 468 | Cortinariaceae | | *Cortinarius* | Cortinarius_sp. (81) | | 28 | 4 | 0 | 0 | | 0 | | 1 | | 0 | | 1 | | 0 | 2 |
| OTU | 485 | Cortinariaceae | | *Cortinarius* | Cortinarius_delibutus (100) | | 16 | 2 | 1 | 0 | | 0 | | 0 | | 0 | | 1 | | 0 | 0 |
| OTU | 496 | Cortinariaceae | | *Cortinarius* | unclassified | | 25 | 2 | 1 | 0 | | 0 | | 0 | | 0 | | 0 | | 0 | 1 |
| OTU | 624 | Cortinariaceae | | *Cortinarius* | unclassified | | 30 | 8 | 1 | 2 | | 0 | | 2 | | 0 | | 0 | | 0 | 3 |
| OTU | 646 | Cortinariaceae | | *Cortinarius* | Cortinarius_colus (95) | | 25 | 7 | 1 | 1 | | 0 | | 1 | | 1 | | 0 | | 2 | 1 |
| OTU | 701 | Cortinariaceae | | *Cortinarius* | unclassified | | 22 | 7 | 0 | 0 | | 0 | | 0 | | 2 | | 0 | | 3 | 2 |
| OTU | 705 | Cortinariaceae | | *Cortinarius* | unclassified | | 1 | 1 | 0 | 0 | | 1 | | 0 | | 0 | | 0 | | 0 | 0 |
| OTU | 762 | Cortinariaceae | | *Cortinarius* | unclassified | | 48 | 2 | 0 | 0 | | 0 | | 1 | | 1 | | 0 | | 0 | 0 |
| OTU | 915 | Cortinariaceae | | *Cortinarius* | Cortinarius_anomalus (95) | | 7 | 2 | 0 | 0 | | 0 | | 0 | | 0 | | 0 | | 1 | 1 |
| OTU | 918 | Cortinariaceae | | *Cortinarius* | Cortinarius_eburneus (75) | | 10 | 2 | 0 | 1 | | 0 | | 0 | | 0 | | 1 | | 0 | 0 |
| OTU | 989 | Cortinariaceae | | *Cortinarius* | unculturedfungus (100) | | 9 | 2 | 0 | 1 | | 0 | | 0 | | 0 | | 0 | | 1 | 0 |
| OTU | 1023 | Cortinariaceae | | *Cortinarius* | Cortinarius_eburneus (60) | | 23 | 5 | 2 | 0 | | 0 | | 0 | | 2 | | 1 | | 0 | 0 |
| OTU | 1142 | Cortinariaceae | | *Cortinarius* | unculturedfungus (74) | | 12 | 2 | 0 | 0 | | 0 | | 0 | | 0 | | 0 | | 2 | 0 |
| OTU | 1238 | Cortinariaceae | | *Cortinarius* | unclassified | | 14 | 6 | 1 | 0 | | 1 | | 1 | | 0 | | 0 | | 3 | 0 |
| OTU | 1348 | Cortinariaceae | | *Cortinarius* | unculturedfungus (95) | | 2 | 1 | 0 | 0 | | 0 | | 0 | | 0 | | 0 | | 1 | 0 |
| OTU | 1577 | Cortinariaceae | | *Cortinarius* | unculturedfungus (98) | | 18 | 3 | 0 | 0 | | 0 | | 1 | | 0 | | 0 | | 2 | 0 |
| OTU | 1606 | Cortinariaceae | | *Cortinarius* | unclassified | | 45 | 4 | 0 | 0 | | 0 | | 1 | | 0 | | 0 | | 1 | 2 |
| OTU | 1904 | Cortinariaceae | | *Cortinarius* | unclassified | | 1 | 1 | 1 | 0 | | 0 | | 0 | | 0 | | 0 | | 0 | 0 |
| OTU | 2097 | Cortinariaceae | | *Cortinarius* | unculturedfungus (94) | | 9 | 2 | 0 | 1 | | 0 | | 0 | | 1 | | 0 | | 0 | 0 |
| OTU | 2392 | Cortinariaceae | | *Cortinarius* | unclassified | | 10 | 3 | 1 | 1 | | 0 | | 0 | | 0 | | 1 | | 0 | 0 |
| OTU | 2593 | Cortinariaceae | | *Cortinarius* | Cortinarius_sp. (62) | | 3 | 2 | 0 | 0 | | 0 | | 1 | | 0 | | 0 | | 0 | 1 |
| OTU | 2944 | Cortinariaceae | | *Cortinarius* | unclassified | | 7 | 3 | 0 | 0 | | 0 | | 1 | | 0 | | 0 | | 1 | 1 |
| OTU | 1186 | Cortinariaceae | | *Inocybe* | Inocybe_lilacina (100) | | 26 | 2 | 0 | 1 | | 0 | | 0 | | 0 | | 1 | | 0 | 0 |
| OTU | 1875 | Cortinariaceae | | *Inocybe* | Inocybe_praetervisa (91) | | 2 | 2 | 0 | 0 | | 0 | | 0 | | 0 | | 2 | | 0 | 0 |
| OTU | 318 | Entolomataceae | | *Entoloma* | Entoloma_sp. (94) | | 15 | 8 | 0 | 0 | | 0 | | 0 | | 3 | | 2 | | 1 | 2 |
| OTU | 635 | Entolomataceae | | *Entoloma* | Entoloma_cuspidiferum (94) | | 9 | 5 | 1 | 1 | | 0 | | 1 | | 1 | | 0 | | 1 | 0 |
| OTU | 869 | Entolomataceae | | *Entoloma* | Entoloma_sp_CDW71 (85) | | 6 | 2 | 0 | 0 | | 1 | | 0 | | 0 | | 0 | | 0 | 1 |
| OTU | 1677 | Entolomataceae | | *Entoloma* | Entoloma_turbidum (100) | | 2 | 2 | 1 | 0 | | 0 | | 0 | | 0 | | 1 | | 0 | 0 |
| OTU | 1141 | Gomphidiaceae | | *Chroogomphus* | Chroogomphus_vinicolor (100) | | 7 | 3 | 0 | 0 | | 1 | | 0 | | 0 | | 0 | | 2 | 0 |
| OTU | 1922 | Gomphidiaceae | | *Gomphidius* | Gomphidius_sp. (98) | | 3 | 1 | 0 | 0 | | 0 | | 0 | | 1 | | 0 | | 0 | 0 |
| OTU | 362 | Hydnaceae | | *Hydnum* | Hydnum_sp. (100) | | 39 | 2 | 0 | 0 | | 1 | | 0 | | 0 | | 0 | | 1 | 0 |
| OTU | 158 | Hygrophoraceae | | *Hygrophorus* | unculturedfungus (100) | | 164 | 4 | 1 | 1 | | 0 | | 0 | | 1 | | 1 | | 0 | 0 |
| OTU | 219 | Hygrophoraceae | | *Hygrophorus* | Hygrophorus_pudorinus (100) | | 177 | 2 | 1 | 0 | | 0 | | 0 | | 0 | | 1 | | 0 | 0 |
| OTU | 1067 | Hysterangiaceae | | *Hysterangium* | Hysterangium_crassirhachis (95) | | 3 | 2 | 1 | 0 | | 0 | | 0 | | 0 | | 1 | | 0 | 0 |
| OTU | 1872 | Hysterangiaceae | | *Hysterangium* | Hysterangium_crassirhachis (98) | | 8 | 5 | 0 | 1 | | 0 | | 0 | | 0 | | 3 | | 1 | 0 |
| OTU | 607 | Paxillaceae | | *Alpova* | unculturedfungus (88) | | 18 | 5 | 0 | 0 | | 0 | | 1 | | 0 | | 1 | | 2 | 1 |
| OTU | 1425 | Rhizopogonaceae | | *Rhizopogon* | Rhizopogon_sp. (100) | | 5 | 3 | 0 | 0 | | 0 | | 0 | | 0 | | 0 | | 3 | 0 |
| OTU | 366 | Russulaceae | | *Lactarius* | Lactarius_rufus (69) | | 31 | 3 | 1 | 0 | | 0 | | 0 | | 2 | | 0 | | 0 | 0 |
| OTU | 595 | Russulaceae | | *Lactarius* | Lactarius_caespitosus (64) | | 8 | 1 | 0 | 0 | | 0 | | 0 | | 0 | | 1 | | 0 | 0 |
| OTU | 1418 | Russulaceae | | *Lactarius* | Lactarius_deceptivus (100) | | 340 | 16 | 2 | 2 | | 0 | | 0 | | 5 | | 4 | | 1 | 2 |
| OTU | 1757 | Russulaceae | | *Lactarius* | Lactarius_deceptivus (100) | | 123 | 12 | 1 | 1 | | 1 | | 1 | | 3 | | 3 | | 1 | 1 |
| OTU | 1813 | Russulaceae | | *Lactarius* | Lactarius_deceptivus (100) | | 865 | 18 | 2 | 2 | | 1 | | 1 | | 5 | | 5 | | 1 | 1 |
| OTU | 2057 | Russulaceae | | *Lactarius* | Lactarius_deceptivus (100) | | 2006 | 20 | 2 | 3 | | 1 | | 1 | | 6 | | 4 | | 2 | 1 |
| OTU | 2058 | Russulaceae | | *Lactarius* | Lactarius_deceptivus (100) | | 45 | 6 | 0 | 1 | | 0 | | 0 | | 2 | | 2 | | 0 | 1 |
| OTU | 2133 | Russulaceae | | *Lactarius* | Lactarius_deceptivus (100) | | 18 | 4 | 0 | 1 | | 0 | | 0 | | 1 | | 2 | | 0 | 0 |
| OTU | 2188 | Russulaceae | | *Lactarius* | Lactarius_deceptivus (100) | | 2 | 2 | 0 | 1 | | 0 | | 0 | | 0 | | 1 | | 0 | 0 |
| OTU | 2686 | Russulaceae | | *Lactarius* | Lactarius_deceptivus (100) | | 4 | 2 | 0 | 0 | | 0 | | 0 | | 1 | | 1 | | 0 | 0 |
| OTU | 11 | Russulaceae | | *Russula* | Russula_sp_F14064 (91) | | 2156 | 9 | 1 | 3 | | 0 | | 1 | | 2 | | 2 | | 0 | 0 |
| OTU | 22 | Russulaceae | | *Russula* | Russula_xerampelina_var_xerampelina (70) | | 1257 | 15 | 2 | 3 | | 1 | | 2 | | 3 | | 3 | | 1 | 0 |
| OTU | 25 | Russulaceae | | *Russula* | Russula_turci (100) | | 1319 | 14 | 2 | 2 | | 1 | | 2 | | 3 | | 4 | | 0 | 0 |
| OTU | 98 | Russulaceae | | *Russula* | unclassified | | 386 | 2 | 1 | 0 | | 0 | | 0 | | 1 | | 0 | | 0 | 0 |
| OTU | 124 | Russulaceae | | *Russula* | Russula_bicolor (100) | | 404 | 15 | 0 | 4 | | 1 | | 1 | | 2 | | 4 | | 2 | 1 |
| OTU | 431 | Russulaceae | | *Russula* | unclassified | | 49 | 2 | 1 | 0 | | 0 | | 0 | | 1 | | 0 | | 0 | 0 |
| OTU | 620 | Russulaceae | | *Russula* | Russula_nigricans (100) | | 7 | 2 | 0 | 0 | | 0 | | 0 | | 0 | | 2 | | 0 | 0 |
| OTU | 1716 | Russulaceae | | *Russula* | Russula_bicolor (96) | | 3 | 2 | 0 | 1 | | 0 | | 0 | | 0 | | 1 | | 0 | 0 |
| OTU | 2038 | Russulaceae | | *Russula* | Russula_bicolor (98) | | 5 | 3 | 0 | 1 | | 0 | | 0 | | 1 | | 0 | | 1 | 0 |
| OTU | 3080 | Russulaceae | | *Russula* | Russula_turci (100) | | 4 | 2 | 1 | 0 | | 0 | | 0 | | 1 | | 0 | | 0 | 0 |
| OTU | 861 | Russulaceae | | *Russula* | unclassified | | 10 | 3 | 1 | 0 | | 0 | | 1 | | 1 | | 0 | | 0 | 0 |
| OTU | 1957 | Russulaceae | | *Russula* | Russula_firmula (63) | | 6 | 2 | 0 | 0 | | 0 | | 0 | | 0 | | 1 | | 1 | 0 |
| OTU | 55 | Russulaceae | | *unidentified* | unculturedLactarius (89) | | 596 | 19 | 2 | 1 | | 3 | | 3 | | 3 | | 4 | | 1 | 2 |
| OTU | 536 | Sclerodermataceae | | *Scleroderma* | Scleroderma_sp. (100) | | 34 | 4 | 0 | 1 | | 0 | | 2 | | 0 | | 0 | | 0 | 1 |
| OTU | 601 | Sebacinaceae | | *Sebacina* | Sebacina_incrustans (100) | | 17 | 2 | 0 | 1 | | 0 | | 0 | | 0 | | 1 | | 0 | 0 |
| OTU | 1923 | Sebacinaceae | | *Sebacina* | Sebacina_sp_RT_2012 (69) | | 52 | 2 | 1 | 0 | | 0 | | 0 | | 1 | | 0 | | 0 | 0 |
| OTU | 272 | Sebacinaceae | | *Sebacina* | Sebacina_sp_RT_2012 (95) | | 134 | 12 | 3 | 2 | | 0 | | 0 | | 3 | | 4 | | 0 | 0 |
| OTU | 36 | Sebacinaceae | | *Sebacina* | Sebacina_sp_src723 (99) | | 570 | 4 | 0 | 1 | | 0 | | 0 | | 1 | | 2 | | 0 | 0 |
| OTU | 327 | Sebacinaceae | | *unidentified* | unculturedSebacina (93) | | 81 | 7 | 0 | 0 | | 0 | | 1 | | 0 | | 2 | | 2 | 2 |
| OTU | 395 | Sebacinaceae | | *unidentified* | unculturedSebacina (93) | | 19 | 6 | 0 | 1 | | 0 | | 1 | | 1 | | 1 | | 1 | 1 |
| OTU | 402 | Sebacinaceae | | *unidentified* | unculturedSebacina_mycobiont_of_Riccardia_palmata (100) | | 46 | 2 | 0 | 0 | | 0 | | 1 | | 0 | | 0 | | 1 | 0 |
| OTU | 585 | Sebacinaceae | | *unidentified* | unculturedSebacina (95) | | 19 | 4 | 0 | 1 | | 0 | | 0 | | 0 | | 1 | | 1 | 1 |
| OTU | 1006 | Sebacinaceae | | *unidentified* | unculturedSebacina_mycobiont_of_Riccardia_latifrons (100) | | 1 | 1 | 0 | 0 | | 0 | | 1 | | 0 | | 0 | | 0 | 0 |
| OTU | 1061 | Sebacinaceae | | *unidentified* | unclassified | | 55 | 5 | 0 | 1 | | 0 | | 1 | | 0 | | 1 | | 1 | 1 |
| OTU | 1303 | Sebacinaceae | | *unidentified* | unculturedSebacina_mycobiont_of_Aneura_pinguis (98) | | 1 | 1 | 0 | 1 | | 0 | | 0 | | 0 | | 0 | | 0 | 0 |
| OTU | 393 | Sebacinaceae | | *unidentified* | unclassified | | 124 | 8 | 1 | 1 | | 1 | | 1 | | 1 | | 2 | | 1 | 0 |
| OTU | 2997 | Sebacinaceae | | *unidentified* | unclassified | | 84 | 8 | 1 | 0 | | 2 | | 0 | | 1 | | 2 | | 2 | 0 |
| OTU | 1808 | Sebacinaceae | | *unidentified* | unculturedSebacina (62) | | 23 | 2 | 1 | 0 | | 0 | | 0 | | 0 | | 1 | | 0 | 0 |
| OTU | 2817 | Sebacinaceae | | *unidentified* | unclassified | | 10 | 5 | 1 | 0 | | 1 | | 0 | | 1 | | 0 | | 2 | 0 |
| OTU | 685 | Sebacinaceae | | *unidentified* | unclassified | | 7 | 4 | 0 | 0 | | 0 | | 0 | | 0 | | 2 | | 2 | 0 |
| OTU | 1563 | Sebacinaceae | | *unidentified* | unculturedSebacina_mycobiont_of_Riccardia_palmata (97) | | 5 | 3 | 0 | 0 | | 3 | | 0 | | 0 | | 0 | | 0 | 0 |
| OTU | 104 | Sebacinaceae | | *unidentified* | unculturedSebacina (92) | | 297 | 21 | 1 | 1 | | 5 | | 2 | | 2 | | 3 | | 4 | 3 |
| OTU | 39 | Sebacinaceae | | *unidentified* | Sebacinaceae_sp_W53 (98) | | 543 | 8 | 0 | 2 | | 1 | | 1 | | 0 | | 1 | | 2 | 1 |
| OTU | 186 | Sebacinaceae | | *unidentified* | unculturedSebacina (92) | | 157 | 3 | 0 | 0 | | 1 | | 0 | | 0 | | 0 | | 1 | 1 |
| OTU | 20 | Sistotremataceae | | *Sistotrema* | unculturedfungus (88) | | 1012 | 24 | 5 | 4 | | 0 | | 2 | | 6 | | 5 | | 1 | 1 |
| OTU | 24 | Sistotremataceae | | *Sistotrema* | unculturedfungus (99) | | 1085 | 7 | 0 | 1 | | 1 | | 2 | | 0 | | 0 | | 1 | 2 |
| OTU | 305 | Sistotremataceae | | *Sistotrema* | Sistotrema_sp. (83) | | 5 | 1 | 0 | 0 | | 0 | | 0 | | 1 | | 0 | | 0 | 0 |
| OTU | 1373 | Sistotremataceae | | *Sistotrema* | Sistotrema_sp. (99) | | 4 | 3 | 0 | 0 | | 0 | | 0 | | 2 | | 1 | | 0 | 0 |
| OTU | 87 | Suillaceae | | *Suillus* | Suillus_sp. (100) | | 325 | 4 | 0 | 0 | | 0 | | 1 | | 0 | | 1 | | 2 | 0 |
| OTU | 215 | Suillaceae | | *Suillus* | Suillus_sp. (71) | | 453 | 18 | 1 | 1 | | 1 | | 5 | | 2 | | 2 | | 2 | 4 |
| OTU | 234 | Suillaceae | | *Suillus* | Suillus_sp. (100) | | 82 | 7 | 1 | 0 | | 1 | | 2 | | 1 | | 1 | | 0 | 1 |
| OTU | 363 | Suillaceae | | *Suillus* | Suillus_sp. (100) | | 106 | 7 | 0 | 0 | | 2 | | 2 | | 0 | | 1 | | 0 | 2 |
| OTU | 1985 | Suillaceae | | *Suillus* | unclassified | | 3 | 2 | 0 | 0 | | 1 | | 0 | | 0 | | 1 | | 0 | 0 |
| OTU | 2819 | Suillaceae | | *Suillus* | unclassified | | 9 | 3 | 0 | 0 | | 0 | | 1 | | 0 | | 0 | | 2 | 0 |
| OTU | 2853 | Suillaceae | | *Suillus* | Suillus_sp. (100) | | 10 | 5 | 0 | 0 | | 2 | | 2 | | 0 | | 1 | | 0 | 0 |
| OTU | 321 | Thelephoraceae | | *Pseudotomentella* | Pseudotomentella_sp. (93) | | 42 | 3 | 0 | 0 | | 0 | | 1 | | 1 | | 1 | | 0 | 0 |
| OTU | 409 | Thelephoraceae | | *Pseudotomentella* | unclassified | | 4 | 2 | 0 | 0 | | 0 | | 1 | | 0 | | 0 | | 0 | 1 |
| OTU | 1692 | Thelephoraceae | | *Pseudotomentella* | unclassified | | 1 | 1 | 0 | 1 | | 0 | | 0 | | 0 | | 0 | | 0 | 0 |
| OTU | 73 | Thelephoraceae | | *Thelephora* | Thelephora_terrestris (98) | | 525 | 8 | 0 | 1 | | 2 | | 1 | | 0 | | 0 | | 3 | 1 |
| OTU | 9 | Thelephoraceae | | *Tomentella* | Tomentella_sublilacina (100) | | 2215 | 3 | 1 | 0 | | 0 | | 0 | | 1 | | 0 | | 1 | 0 |
| OTU | 344 | Thelephoraceae | | *Tomentella* | unclassified | | 33 | 4 | 0 | 2 | | 0 | | 0 | | 0 | | 2 | | 0 | 0 |
| OTU | 423 | Thelephoraceae | | *Tomentella* | Tomentella_sp_4_RT_2012 (78) | | 27 | 4 | 0 | 0 | | 1 | | 0 | | 2 | | 0 | | 1 | 0 |
| OTU | 824 | Thelephoraceae | | *Tomentella* | Tomentella_bryophila (77) | | 18 | 2 | 0 | 0 | | 0 | | 0 | | 0 | | 1 | | 0 | 1 |
| OTU | 1743 | Thelephoraceae | | *Tomentella* | Tomentella_badia (95) | | 2 | 2 | 1 | 0 | | 0 | | 0 | | 1 | | 0 | | 0 | 0 |
| OTU | 1989 | Thelephoraceae | | *Tomentella* | Tomentella_sp_EL274 (100) | | 1 | 1 | 0 | 0 | | 0 | | 0 | | 0 | | 1 | | 0 | 0 |
| OTU | 2461 | Thelephoraceae | | *Tomentella* | Tomentella_sublilacina (98) | | 7 | 2 | 1 | 0 | | 0 | | 0 | | 1 | | 0 | | 0 | 0 |
| OTU | 199 | Thelephoraceae | | *Tomentellopsis* | unclassified | | 22 | 7 | 3 | 0 | | 0 | | 1 | | 1 | | 1 | | 1 | 0 |
| OTU | 2190 | Thelephoraceae | | *Tomentellopsis* | unclassified | | 84 | 5 | 3 | 0 | | 0 | | 1 | | 1 | | 0 | | 0 | 0 |
| OTU | 2574 | Thelephoraceae | | *Tomentellopsis* | Tomentellopsis_sp. (100) | | 1 | 1 | 0 | 1 | | 0 | | 0 | | 0 | | 0 | | 0 | 0 |
| OTU | 2720 | Thelephoraceae | | *Tomentellopsis* | unculturedfungus (69) | | 4 | 2 | 1 | 0 | | 0 | | 1 | | 0 | | 0 | | 0 | 0 |
| OTU | 88 | Tricholomataceae | | *Tricholoma* | unclassified | | 36 | 2 | 0 | 1 | | 0 | | 0 | | 1 | | 0 | | 0 | 0 |
| OTU | 169 | Tricholomataceae | | *Tricholoma* | Tricholoma_sp. (94) | | 158 | 3 | 0 | 1 | | 0 | | 0 | | 0 | | 2 | | 0 | 0 |
| OTU | 195 | Tricholomataceae | | *Tricholoma* | Tricholoma_sp_RT_2012 (100) | | 26 | 2 | 0 | 0 | | 0 | | 1 | | 0 | | 0 | | 0 | 1 |
| OTU | 1263 | Tricholomataceae | | *Tricholoma* | Tricholoma_focale (74) | | 3 | 2 | 0 | 0 | | 0 | | 0 | | 1 | | 1 | | 0 | 0 |
| Ascomycota - Dothideomycetes | | | | | | | | | | | | | | | | | | | | | |
| OTU | 41 | Incertae_sedis | *Cenococcum* | | unculturedCenococcum (100) | 770 | | 31 | 4 | 2 | 3 | | 2 | | 3 | | 6 | | 5 | | 6 |
| OTU | 130 | Incertae_sedis | *Cenococcum* | | unculturedCenococcum (100) | 317 | | 22 | 3 | 4 | 2 | | 2 | | 4 | | 4 | | 2 | | 1 |
| OTU | 146 | Incertae_sedis | *Cenococcum* | | Cenococcum_geophilum (78) | 135 | | 33 | 3 | 5 | 6 | | 4 | | 4 | | 3 | | 3 | | 5 |
| OTU | 152 | Incertae_sedis | *Cenococcum* | | unculturedCenococcum (73) | 320 | | 28 | 6 | 4 | 4 | | 2 | | 5 | | 3 | | 3 | | 1 |
| OTU | 278 | Incertae_sedis | *Cenococcum* | | unculturedCenococcum (70) | 39 | | 11 | 3 | 0 | 1 | | 2 | | 3 | | 0 | | 1 | | 1 |
| OTU | 774 | Incertae_sedis | *Cenococcum* | | Cenococcum_geophilum (96) | 16 | | 6 | 2 | 1 | 0 | | 1 | | 1 | | 1 | | 0 | | 0 |
| OTU | 1585 | Incertae_sedis | *Cenococcum* | | Cenococcum_geophilum (100) | 10 | | 3 | 0 | 2 | 0 | | 1 | | 0 | | 0 | | 0 | | 0 |
| OTU | 1669 | Incertae_sedis | *Cenococcum* | | Cenococcum_geophilum (92) | 70 | | 11 | 2 | 3 | 1 | | 0 | | 1 | | 2 | | 2 | | 0 |
| OTU | 1708 | Incertae_sedis | *Cenococcum* | | Cenococcum_geophilum (93) | 3 | | 3 | 1 | 1 | 1 | | 0 | | 0 | | 0 | | 0 | | 0 |
| OTU | 2177 | Incertae_sedis | *Cenococcum* | | Cenococcum_geophilum (100) | 15 | | 7 | 3 | 0 | 0 | | 1 | | 2 | | 1 | | 0 | | 0 |
| Ascomycota - Leotiomycetes | | |  | |  |  | |  |  |  |  | |  | |  | |  | |  | |  |
| OTU | 504 | Incertae_sedis | *Cadophora* | | unclassified | 42 | | 3 | 0 | 1 | 0 | | 1 | | 0 | | 0 | | 0 | | 1 |
| OTU | 1233 | Incertae_sedis | *Cadophora* | | unclassified | 17 | | 4 | 1 | 2 | 0 | | 0 | | 1 | | 0 | | 0 | | 0 |
| OTU | 1800 | Incertae_sedis | *Cadophora* | | unculturedfungus (95) | 6 | | 6 | 0 | 3 | 0 | | 0 | | 1 | | 1 | | 1 | | 0 |
| OTU | 560 | Incertae_sedis | *Cadophora* | | Cadophora_melinii (89) | 11 | | 4 | 0 | 0 | 0 | | 1 | | 0 | | 1 | | 1 | | 1 |
| OTU | 416 | Incertae_sedis | *Cadophora* | | Cadophora_sp_5R24_1 (96) | 53 | | 12 | 1 | 1 | 0 | | 1 | | 3 | | 4 | | 1 | | 1 |
| OTU | 1494 | Incertae_sedis | *Cadophora* | | Cadophora_sp_AU_BD06 (90) | 2 | | 1 | 0 | 0 | 0 | | 0 | | 0 | | 0 | | 0 | | 1 |
| OTU | 2627 | Incertae_sedis | *Cadophora* | | Cadophora_sp_MTFA12 (69) | 22 | | 4 | 0 | 1 | 0 | | 0 | | 1 | | 1 | | 0 | | 1 |
| Ascomycota - Pezizomycetes | | |  | |  |  | |  |  |  |  | |  | |  | |  | |  | |  |
| OTU | 240 | Discinaceae | *Hydnotrya* | | Hydnotrya_sp. (98) | 62 | | 3 | 0 | 0 | 1 | | 0 | | 0 | | 0 | | 2 | | 0 |
| OTU | 1000 | Discinaceae | *Hydnotrya* | | Hydnotrya_sp. (98) | 16 | | 3 | 0 | 1 | 1 | | 0 | | 0 | | 0 | | 1 | | 0 |
| OTU | 791 | Pezizaceae | *Peziza* | | Peziza_ammophila (100) | 11 | | 3 | 0 | 0 | 0 | | 0 | | 0 | | 1 | | 1 | | 1 |
| OTU | 2209 | Pezizaceae | *Peziza* | | Peziza_sp_B276 (99) | 2 | | 2 | 0 | 1 | 1 | | 0 | | 0 | | 0 | | 0 | | 0 |
| OTU | 341 | Tuberaceae | *Tuber* | | Tuber_pacificum (100) | 18 | | 4 | 0 | 1 | 1 | | 1 | | 0 | | 0 | | 1 | | 0 |
| OTU | 532 | Tuberaceae | *Tuber* | | unculturedectomycorrhizal_fungus (79) | 14 | | 4 | 0 | 1 | 1 | | 0 | | 0 | | 1 | | 1 | | 0 |
| OTU | 99 | Helvellaceae | *Helvella* | | Helvella_sp_SOC902 (83) | 32 | | 2 | 0 | 0 | 1 | | 0 | | 0 | | 0 | | 1 | | 0 |
| Ascomycota - Sordariomycetes | | |  | |  |  | |  |  |  |  | |  | |  | |  | |  | |  |
| OTU | 2557 | Chaetosphaeriaceae | *Chloridium* | | Chloridium_sp_TMS_2011 (65) | 4 | | 4 | 1 | 0 | 0 | | 0 | | 0 | | 1 | | 2 | | 0 |

**Table S2** Mean and standard error of number of reads, richness and coverage of EM fungal OTUs with ANOVA testing. Different letters indicate significant differences (*P* < 0.05).

**BULK SOIL**

|  | *Picea mariana* | *Abies balsamea* | *Pinus banksiana* | *Pinus mugo* |
| --- | --- | --- | --- | --- |
| Number of reads | 507 ± 106 | 617± 124 | 362 ± 85 | 270 ± 68 |
| Observed OTU richness | 23.8 ± 1.4 (a) | 27.8 ± 3.2 (a) | 20.9 ± 2.7 (ab) | 13.6 ± 2.2 (b) |
| Chao | 34.9 ± 4.4 (ab) | 40.9 ± 5.0 (a) | 37.6 ± 6.1 (a) | 18.4 ± 4.0 (b) |
| Good’s coverage | 0.98 ± 0.01 | 0.98 ± 0.00 | 0.96 ± 0.02 | 0.96 ± 0.03 |
| Rarefied OTU richness * | 15.0 ±1.1 | 16.4± 2.1 | 13.7 ± 1.5 | 11.3 ± 1.5 |
| Rarefied coverage* | 0.94 ± 0.01 | 0.94 ± 0.01 | 0.95 ± 0.01 | 0.97 ± 0.01 |

**ROOT SAMPLE**

|  | *Picea mariana* | *Abies balsamea* | *Pinus banksiana* | *Pinus mugo* |
| --- | --- | --- | --- | --- |
| Number of reads | 1102 ± 335 | 914 ± 330 | 266 ± 84 | 332 ± 85 |
| Observed OTU richness | 20.4 ± 2.1 (a) | 18.0 ± 2.2 (a) | 10.1 ± 1.4 (b) | 14.4± 1.7 (ab) |
| Chao | 26.4 ± 2.7 (a) | 26.4 ± 2.5 (a) | 13.4 ± 1.8 (b) | 21.1 ± 2.9 (ab) |
| Good’s coverage | 0.99 ± 0.00 | 0.99 ± 0.00 | 0.96 ± 0.03 | 0.97 ± 0.01 |
| Rarefied OTU richness * | 11.9 ± 0.9 | 9.4 ± 0.7 | 8.7 ± 1.2 | 10.8 ± 1.3 |
| Rarefied coverage* | 0.97 ± 0,00 | 0.97 ± 0,00 | 0.97 ± 0,01 | 0.96 ± 0,00 |

All values: mean ± standard error.

*Rarefied at 100 reads.

** Different letters indicate significant difference between host species in Tukey HSD test.

|  | Number of OTUs | Number of reads | Genus included |
| --- | --- | --- | --- |
| Russulaceae | 23 | 9,644 | *Lactarius, Russula* |
| Atheliaceae | 22 | 9,257 | *Amphinema, Piloderma, Tylospora* |
| Thelephoraceae | 15 | 2,986 | *Pseudotomentella, Tomentella, Tomentellopsis, Thelephora* |
| Sebacinaceae | 20 | 2,245 | *Sebacina* |
| Cortinariaceae | 39 | 2,244 | *Cortinarius, Inocybe* |
| Sistotremataceae | 4 | 2,106 | *Sistotrema* |
| Incertae sedis | 10 | 1,695 | *Cenococcum* |
| Suillaceae | 7 | 988 | *Suillus* |
| Bolbitiaceae | 4 | 602 | *Alnicola, Hebeloma* |
| Amanitaceae | 3 | 370 | *Amanita* |
| Hygrophoraceae | 2 | 341 | *Hygrophorus* |
| Clavulinaceae | 3 | 319 | *Clavulina* |
| Albatrellaceae | 5 | 285 | *Leucophleps* |
| Tricholomataceae | 4 | 223 | *Tricholoma* |
| Boletaceae | 3 | 179 | *Boletus* |
| Incertae sedis | 7 | 153 | *Cadophora* |
| Discinaceae | 2 | 78 | *Hydnotrya* |
| Hydnaceae | 1 | 39 | *Hydnum* |
| Sclerodermataceae | 1 | 34 | *Scleroderma* |
| Entolomataceae | 4 | 32 | *Entoloma* |
| Tuberaceae | 2 | 32 | *Tuber* |
| Helvellaceae | 1 | 32 | *Helvella* |
| Paxillaceae | 1 | 18 | *Alpova* |
| Cantharellaceae | 3 | 16 | *unidentified* |
| Bankeraceae | 4 | 13 | *Hydnellum, Phellodon, Boletopsis, Sarcodon* |
| Pezizaceae | 2 | 13 | *Peziza* |
| Hysterangiaceae | 2 | 11 | *Hysterangium* |
| Gomphidiaceae | 2 | 10 | *Gomphidius, Chroogomphus* |
| Rhizopogonaceae | 1 | 5 | *Rhizopogon* |
| Ceratobasidiaceae | 2 | 4 | *Ceratobasidium* |
| Chaetosphaeriaceae | 1 | 4 | *Chloridium* |

**Table S3** Total relative abundance of the different EM fungal families encountered in roots and bulk soil samples in terms of number of OTUs and number of reads.

*
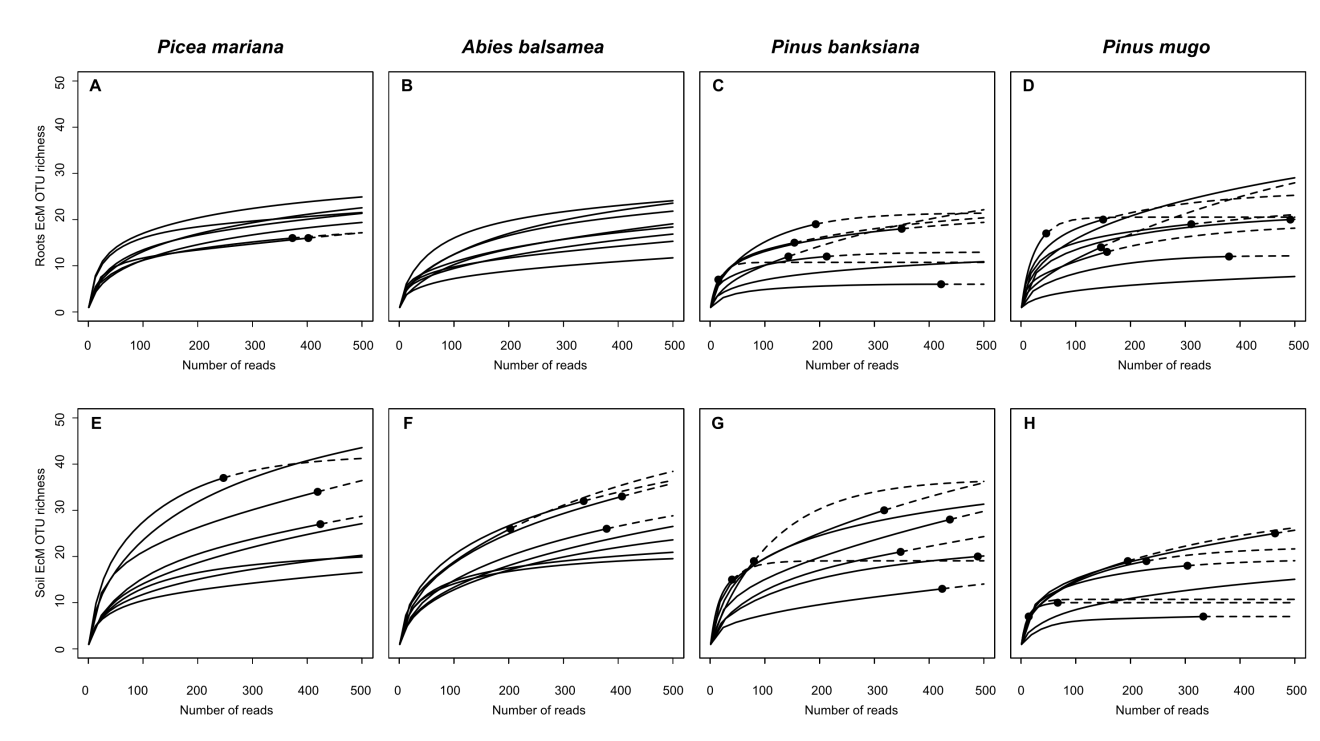
*

**Figure S1** Rarefaction curves of EM fungal OTUs in roots (A-D) and soil (E-H) samples against the number of 454 reads excluding singletons for *Picea mariana* (A, E), *Abies balsamea* (B, F), *Pinus banksiana* (C, G), and *Pinus mugo* (D, H). The dashed section of the curves represents richness extrapolations. The analyses are based on 1000 iterations of re-sampling without replacement.


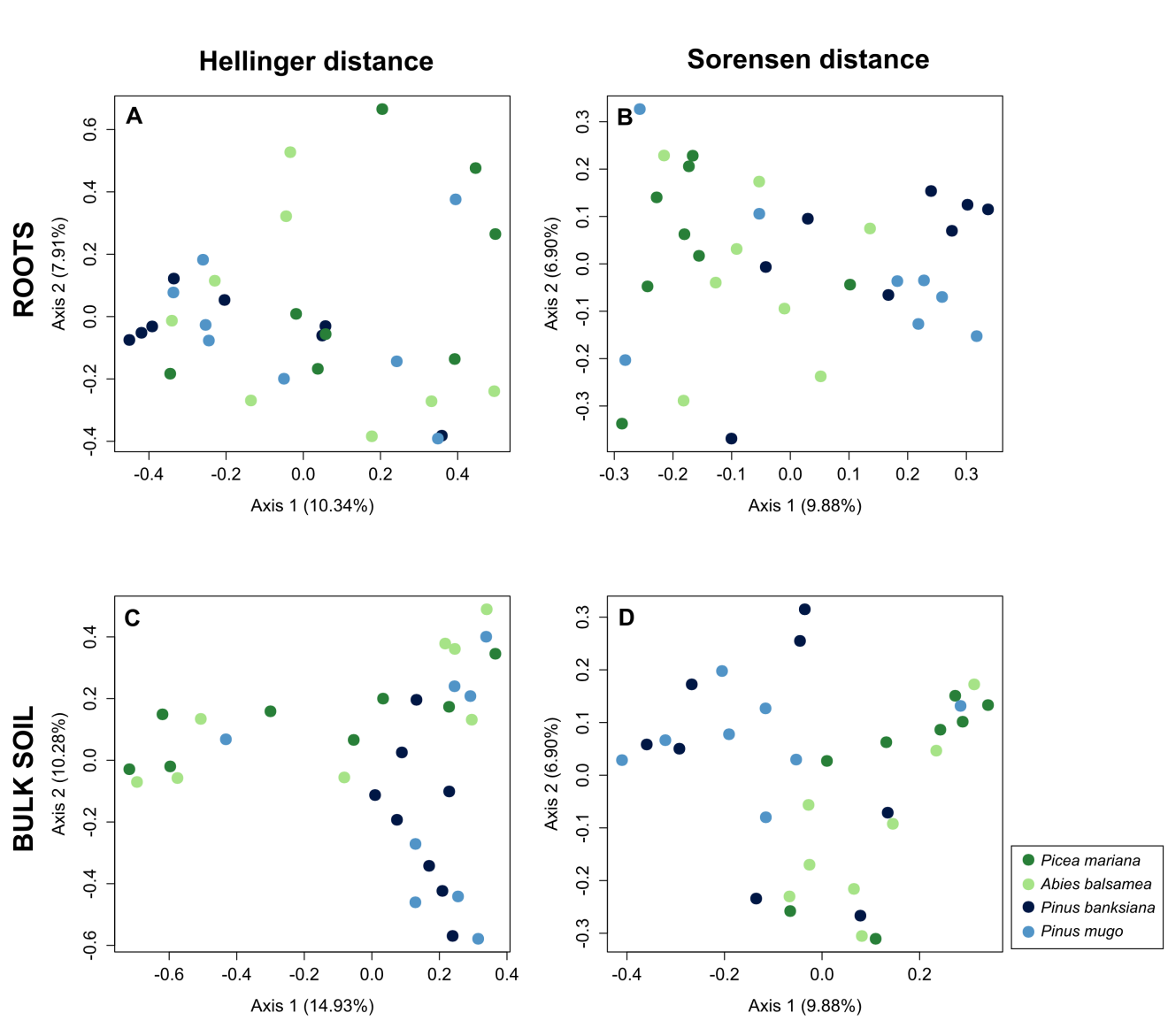


**Figure S2** Principal coordinate analysis (PCoA) of roots (A-B) and bulk soil (C-D) associated EM fungal community based the Hellinger distance (A and C) and the Sorensen (B and D) dissimilarity.


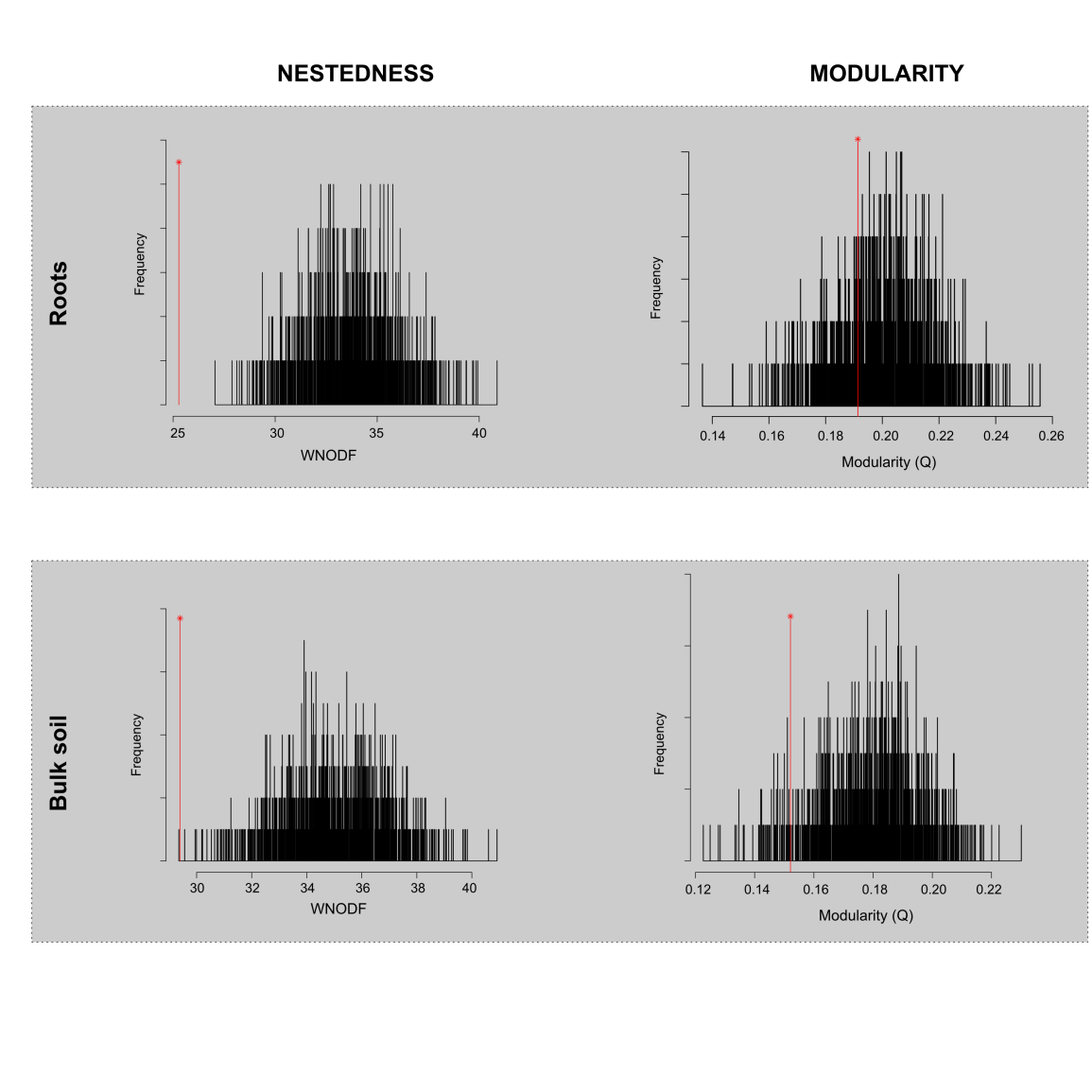
**Figure S3** Modularity and nestedness of roots and bulk soil data in relation to the 1000 matrices generated with a null model preserving rows and columns sums.

**
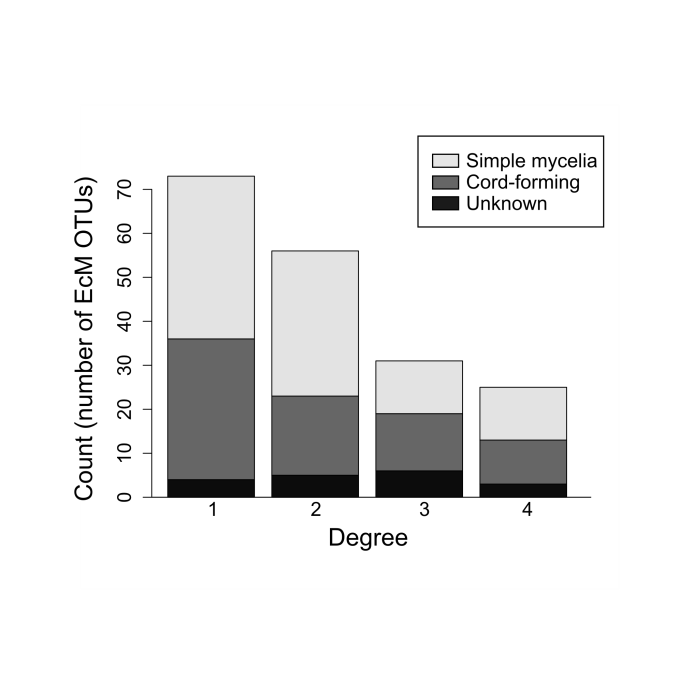
Figure S4** Degree distribution of EM fungal OTUs in function of exploration type.
